# Comparative effects of exercise and nutritional interventions on muscle mass and body composition in adults with cancer: a systematic review and network meta-analysis of randomized controlled trials

**DOI:** 10.3389/or.2026.1926991

**Published:** 2026-10-29

**Authors:** Siqi Gao, Yanting Dong

**Affiliations:** Capital Institute of Physical Education and Sports, Beijing, China

**Keywords:** body composition, cancer, exercise, network meta-analysis, nutrition, randomized controlled trial

## Abstract

**Introduction:**

Loss of muscle mass and changes in body composition are common in adults with cancer. We compared exercise, nutritional, and combined strategies and assessed the certainty and clinical interpretability of the evidence.

**Methods:**

We searched PubMed, Embase, the Cochrane Library, and Web of Science through April 2026 for randomized controlled trials reporting appendicular skeletal muscle mass, skeletal muscle index, lean mass, body mass index, body weight, or fat mass. Eight nodes were evaluated: standard of care (SOC), active low-intensity control (ALC), resistance exercise (RE), multicomponent exercise (ME), nutrition-only intervention (NOI), exercise-nutrition intervention without resistance exercise as the primary component (ENI), resistance exercise plus nutrition-only intervention (RE + NOI), and comprehensive exercise-nutrition intervention incorporating resistance exercise (ENI + RE). Random-effects network meta-analysis produced mean differences (MDs) with 95% confidence intervals (CIs); certainty was assessed with GRADE/CINeMA.

**Results:**

Twenty-one trials and eight intervention nodes were included. Most network estimates were of low certainty. ME was associated with lower BMI than SOC (MD −3.53, 95% CI −7.01 to −0.04), but not with statistically significant differences in body weight or fat mass. ENI + RE was associated with lower lean mass than SOC (MD −6.72, 95% CI −12.43 to −1.02); this sparse, low-certainty estimate should not be interpreted as evidence that combined interventions are harmful or ineffective. No intervention showed a consistent advantage across muscle mass outcomes.

**Conclusion:**

Relative rankings did not establish clinical superiority. Sparse direct evidence, heterogeneous cancer and treatment contexts, varied intervention protocols and measurement methods, and low certainty limit inference. More standardized head-to-head trials are needed.

## 1 Introduction

Changes in body weight and body composition, particularly fat mass and muscle mass, are clinically relevant consequences of cancer and its treatment (1). Exercise and nutritional interventions have therefore gained increasing attention as non-pharmacological strategies to preserve body composition and functional status in patients with cancer. During disease progression and anticancer treatment, patients frequently experience systemic inflammation, inadequate nutritional intake, metabolic disturbances, and reduced physical activity, all of which may contribute to skeletal muscle loss, reduced muscle strength, and impaired physical function (2). Sarcopenia is typically characterized by low muscle strength accompanied by reduced muscle quantity or quality, and, in severe cases, poor physical performance (3). It is common in oncology populations. A meta-analysis including 81,814 patients with solid tumors reported an overall sarcopenia prevalence of approximately 35.3%, with a higher prevalence among patients receiving palliative care (4). Sarcopenia has also been associated with treatment-related toxicity, physical disability, and adverse clinical outcomes (5). These consequences may further increase hospitalizations, readmissions, and healthcare costs, while adding to productivity loss and caregiving burden for patients and their caregivers.

Current interventions used to preserve muscle mass and body composition during and after cancer treatment include exercise training, dietary counselling, oral nutritional supplementation, protein or amino acid supplementation, and combined exercise-nutrition strategies. However, trials vary substantially in intervention type, intensity, duration, outcome selection, and measurement method, and direct comparisons remain limited (6). The ASCO guideline on cancer cachexia notes that dietary counselling, with or without oral nutritional supplementation, may increase body weight in some studies, but the overall evidence remains limited because outcomes and assessment methods are heterogeneous (7). Consequently, the relative effects of these strategies on lean mass, fat mass, body mass index (BMI), body weight, skeletal muscle index (SMI), and appendicular skeletal muscle mass (ASM) in adults with cancer remain uncertain.

Exercise and nutritional interventions may improve skeletal muscle mass, body weight, and physical function in patients with cancer-related sarcopenia by stimulating muscle protein synthesis, modulating inflammation and metabolic disturbances, increasing energy and protein intake, and attenuating disuse-related muscle atrophy. The ESPEN guideline on nutrition in cancer recommends that patients with cancer maintain or increase physical activity to support muscle mass, physical function, and metabolic status, and emphasizes that nutritional care should be integrated with exercise training (8). However, current guidelines do not provide clear and consistent recommendations regarding the optimal type, intensity, or frequency of exercise, the type or dose of nutritional supplementation, or the most appropriate combined intervention strategy. Moreover, existing trials continue to differ in intervention protocols and outcome measurement approaches.

Several systematic reviews and meta-analyses have examined interventions targeting sarcopenia-related outcomes in patients with cancer. Zhao et al. included 59 randomized controlled trials and found that exercise, nutritional, and multicomponent interventions may improve muscle mass, muscle strength, and some measures of physical function in adults with cancer. However, their analysis primarily emphasized SUCRA-based rankings, and the limited assessment of evidence certainty reduced the clinical interpretability of the findings (9). Liu et al. further compared different exercise modalities in patients with cancer-related sarcopenia and suggested that resistance exercise may have advantages in improving lean mass and leg extension function. Nevertheless, that review focused mainly on exercise interventions and did not evaluate dietary counselling, oral nutritional supplementation, protein or amino acid supplementation, or combined exercise–nutrition interventions as distinct intervention categories. Although risk of bias, consistency, and publication bias were assessed, the certainty of evidence from the network meta-analysis was not systematically evaluated (10).

Previous reviews have therefore been limited by broad intervention classifications, inconsistent outcome measurement, insufficient certainty-of-evidence assessment, and limited interpretation of clinical relevance. Network meta-analysis can integrate direct and indirect evidence and compare several interventions simultaneously, but its validity depends on connected networks, plausible transitivity, and adequate direct evidence. To reduce the risk of conclusions based on rankings alone, we interpreted SUCRA alongside effect estimates, confidence intervals, network geometry, and GRADE/CINeMA certainty.

## 2 Materials and methods

This network meta-analysis (NMA) was conducted and reported in accordance with the Preferred Reporting Items for Systematic Reviews and Meta-Analyses extension statement for network meta-analyses (PRISMA-NMA; Supplementary Table S1) (11). The protocol was prospectively registered in PROSPERO (CRD420261438956).

### 2.1 Data sources and search strategy

A systematic literature search was conducted in PubMed, Embase, the Cochrane Library, and Web of Science. The search strategy combined terms related to cancer, sarcopenia, muscle mass and function, randomized controlled trials, exercise, and nutrition. Search terms included “cancer,” “neoplasia,” “tumor/tumour,” “malignancy,” “carcinoma,” “sarcopenia,” “skeletal muscle index,” “muscle mass,” “lean mass,” “appendicular skeletal muscle,” “handgrip strength,” “physical performance,” “randomized clinical trial,” “sport,” “physical activity,” “exercise training,” “nutrition therapy,” “diet,” “dietary pattern,” “food patterns,” “nutrition,” “protein,” and “oral nutritional supplement” (Supplementary Table S2). Searches were performed from database inception to 1 April 2026. Both free-text terms and controlled vocabulary terms were used, with no language restrictions.

### 2.2 Selection criteria

Studies were eligible if they were randomized controlled trials involving adults with cancer, reported at least one muscle mass or body composition outcome, and evaluated one or more predefined intervention nodes. These nodes included standard of care or usual care (SOC), active low-intensity control (ALC), resistance exercise (RE), multicomponent exercise combining resistance exercise with aerobic or other exercise modalities (ME), nutrition-only intervention (NOI), exercise-plus-nutrition intervention in which resistance exercise was not the primary component (ENI), resistance exercise combined with nutrition-only intervention (RE + NOI), and comprehensive exercise-plus-nutrition intervention incorporating resistance exercise (ENI + RE).

SOC was standard treatment, usual care, or a control condition without additional structured exercise or nutritional intervention. ALC was an active low-intensity condition such as health education, general physical activity advice, stretching, relaxation, or attention control. RE comprised structured exercise against external resistance. ME was defined as multicomponent exercise-only treatment containing at least two modalities (for example, resistance, aerobic, flexibility, balance, or functional exercise) without a nutritional component; the abbreviation ME was retained to preserve continuity with the prespecified analytic node. NOI comprised dietary counselling, oral nutritional supplementation, protein or amino acid supplementation, omega-3 fatty acids, beta-hydroxy-beta-methylbutyrate, or other nutritional support without structured exercise. ENI combined exercise and nutrition when resistance exercise was not clearly the primary exercise component. RE + NOI combined resistance exercise as the primary exercise modality with a nutrition-only intervention. ENI + RE comprised a comprehensive exercise-nutrition intervention explicitly incorporating resistance exercise.

Eligible trials reported at least one of the following outcomes: appendicular skeletal muscle mass (ASM), lean mass, fat mass, body mass index (BMI), body weight, or skeletal muscle index (SMI). These are muscle mass and body composition outcomes; a diagnosis of sarcopenia or sarcopenic obesity was not required for eligibility.

Reports from the same randomized cohort were linked and treated as a single trial; companion reports were retained only when they contributed eligible outcome data not available in the primary report. Studies were excluded if they did not clearly define the relevant outcome measures or were reviews, case reports, conference abstracts, protocols, or non-randomized studies. Records were first screened by title and abstract, followed by full-text assessment. All eligible RCTs were independently checked by two reviewers to ensure that the most recent and complete data were included.

### 2.3 Data extraction and risk-of-bias assessment

Two reviewers independently extracted data from the included RCTs using a standardized data extraction form. Any discrepancies were resolved through discussion with a third reviewer. The following information was extracted from each study: first author, publication year, sample size, patient age, sex distribution, geographical region, follow-up duration, and intervention protocols for the experimental and control groups.

For continuous outcomes, the mean change from baseline and its standard deviation (SD) were preferentially extracted. When studies reported only baseline and endpoint means and SDs, the mean change was calculated as the difference between the two time points. The SD of the change score was calculated from the baseline SD, endpoint SD, and the within-participant correlation coefficient using the following formula:

$$SD_{\text{change}}=\sqrt{\text{Baseline }SD^{2}+\text{Endpoint }SD^{2}-2R\times \text{Baseline SD}\times \text{Endpoint SD}}$$

where (R) represents the within-participant correlation between baseline and endpoint measurements. If (R) could not be derived from an individual study, it was imputed using the correlation coefficient derived from the trial with the largest sample size, the lowest risk of bias, and available change-score data.

Risk of bias was assessed using the revised Cochrane Risk of Bias tool for randomized trials (RoB 2), covering the randomization process, deviations from intended interventions, missing outcome data, measurement of the outcome, and selection of the reported result. Each domain and the overall judgment were rated as low risk, some concerns, or high risk (12).

### 2.4 Statistical analysis

Network meta-analysis was performed using Stata 17.0 MP. For continuous outcomes measured using the same scale and unit across studies, treatment effects were expressed as mean differences (MDs) with 95% confidence intervals (CIs). Multi-arm trials were expanded into pairwise comparisons using an augmented data format, while preserving the within-study correlation structure during estimation to avoid underestimation of standard errors resulting from repeated use of the same control group.

The primary analysis was conducted under the consistency assumption using a random-effects model, with the between-study variance (\tau^2^) estimated by restricted maximum likelihood (REML). When closed loops were present in the network, global inconsistency was assessed using the design-by-treatment interaction model, and local inconsistency was evaluated using the node-splitting method. A (P) value < 0.05 was considered indicative of potential inconsistency. Loop-specific inconsistency was further assessed using the inconsistency factor (IF); if the 95% CI of the IF included zero, there was no statistical evidence of disagreement between direct and indirect evidence.

Network plots were generated to visualize the geometry of each treatment network. In the network graphs, node size was proportional to the total sample size for each intervention, and the thickness of connecting lines reflected the number of studies directly comparing the connected interventions.

To rank interventions, we used the surface under the cumulative ranking curve (SUCRA), probability of being ranked best, and mean rank. Rankings were treated as descriptive relative orderings and were interpreted only alongside effect sizes, 95% CIs, and certainty of evidence. Comparison-adjusted funnel plots were prespecified only for outcome networks with at least 10 studies. Leave-one-out sensitivity analyses sequentially excluded each study and reran the random-effects consistency model.

Because the outcome networks were sparse and contained too few studies for credible covariate adjustment, network meta-regression and formal subgroup analyses were not performed in the revised analysis. Potential effect modifiers--cancer type and treatment setting, exercise modality and intensity, intervention duration, nutritional supplement type, baseline age and nutritional status, and body-composition measurement method--were instead evaluated qualitatively when assessing transitivity and interpreting heterogeneity.

### 2.5 Certainty of evidence assessment

The certainty of evidence for the network meta-analysis was assessed using the GRADE framework and CINeMA (Confidence in Network Meta-Analysis). Evidence from randomized controlled trials was initially rated as high certainty. Overall certainty was evaluated across six domains: within-study bias, indirectness, imprecision, heterogeneity, incoherence, and reporting bias, including publication bias and small-study effects.

Within-study bias was assessed with RoB 2 and incorporated at the comparison level through the CINeMA contribution matrix. Indirectness was judged against transitivity and exchangeability, considering cancer type and treatment setting, baseline age and nutritional status, intervention components and intensity, follow-up duration, and outcome measurement method (DXA, BIA, or CT where reported).

No universally validated minimal important difference is available for these body composition outcomes across heterogeneous adult cancer populations. Accordingly, imprecision and clinical relevance were judged from the effect estimate, width of the 95% CI, whether the CI crossed the null, and whether plausible effects would change clinical interpretation. The pragmatic thresholds specified in the protocol were used only as sensitivity aids and not as definitive criteria for benefit.

Heterogeneity was primarily judged using the between-study variance estimated from the random-effects model and by considering the position of the prediction interval relative to the prespecified MID. For networks containing closed loops, agreement between direct and indirect evidence was assessed using the local node-splitting method or the SIDE approach, together with the design-by-treatment interaction model for global inconsistency. For networks without closed loops, incoherence could not be estimated and was recorded as not estimable in CINeMA.

Reporting bias was assessed from trial registration, selective reporting concerns, and the availability of grey literature. Comparison-adjusted funnel plots were not generated because no outcome network contained at least 10 studies. Each CINeMA domain was rated as no concerns, some concerns, or major concerns, and overall certainty was categorized as high, moderate, low, or very low.

## 3 Results

### 3.1 Study selection and study characteristics

The database search identified 1,223 records. After title/abstract screening and full-text assessment of 59 reports, 21 randomized controlled trials met the eligibility criteria (Figure 1) (13–34).

**FIGURE 1:**
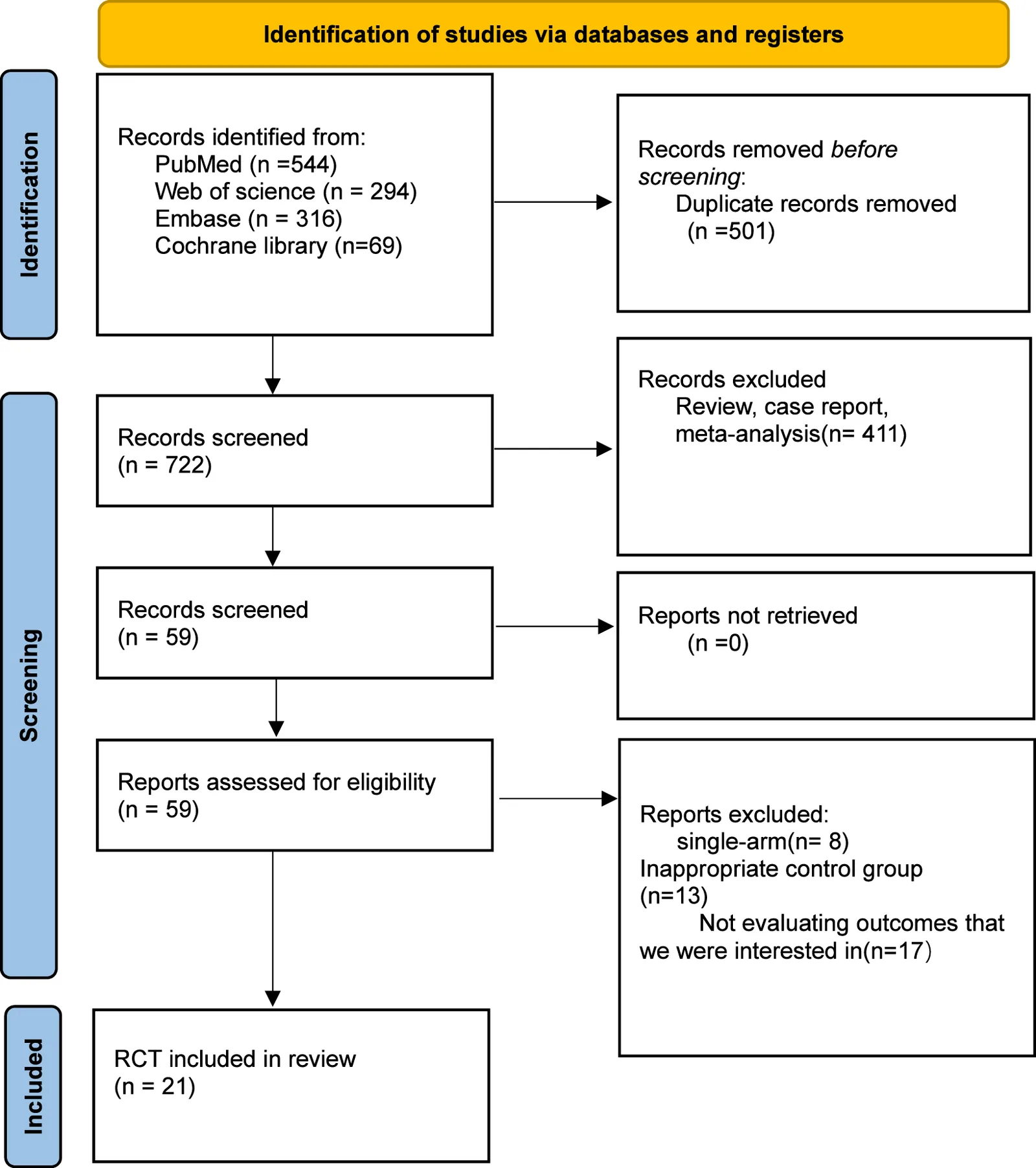
PRISMA flow diagram of study selection.

The 21 randomized trials evaluated eight intervention nodes: SOC, ALC, RE, ME, NOI, ENI, RE + NOI, and ENI + RE. Because individual reports used different outcome-specific analytic populations, a single participant denominator was not applied across networks; each result is therefore described by the number of contributing trials and intervention nodes.

Intervention duration varied across studies. Nutritional supplementation or dietary counselling interventions ranged from 8 days to 6 months, most commonly lasting 8–12 weeks. Resistance exercise alone was delivered for 8 weeks to 12 months. Combined aerobic and resistance exercise interventions generally lasted 7–17 weeks, although some studies extended to 6 months. Multicomponent exercise or prehabilitation interventions typically lasted approximately 12–15 weeks, whereas sport-specific interventions, such as football training, were delivered for up to 32 weeks.

The included studies were conducted across multiple countries and regions, including the United States (n = 4), Australia (n = 3), Canada (n = 2), the United Kingdom (n = 2), Japan (n = 2), and Sweden (n = 2), as well as China, Germany, Denmark, Brazil, the Netherlands, India, and multinational European settings. Most participants were middle-aged or older adults with cancer, with mean ages generally ranging from approximately 50 to 70 years, although some studies included younger or older populations. Sex distribution varied by cancer type, with some studies predominantly involving men with prostate cancer and others predominantly involving women with breast cancer; overall, men accounted for a slightly larger proportion of participants. Post-intervention follow-up durations also varied, most commonly ranging from 8 to 16 weeks, with some studies extending follow-up to 6 months, 12 months, 30 days after surgery, or 1 year after surgery. Detailed characteristics of the included studies are presented in Table 1.

**TABLE 1:** Study characteristics and clinical context of the included randomized trials.

Study	Year	Country	Cancer type/population	Sample size	Male/female	Age (mean ± SD) year	Final follow‐up
Jin Fu	2025	China	Mixed cancers	8/17	12/13	FSMP:57.4 ± 16.0; Ctrl:47.9 ± 13.9	8 weeks
Galvão DA	2010	Australia	Prostate cancer	29/28	57/0	Exp:69.5 ± 7.3; Ctrl: 70.1 ± 7.3	12 weeks
Lam T	2020	Australia	Prostate cancer	10/10	25/0	PRT: 69.3 ± 8.3; Ctrl:71.8 ± 6.2	12 months
Dieli-Conwright CM	2019	USA	Breast cancer	48/49	0/97	Exp:50.3 ± 11.1; Ctrl:50.8 ± 11.0	16 weeks
Grote M	2018	Germany	Head and neck cancer	10/10	5/15	PRT:60.2 ± 4.7; Ctrl:61.5 ± 15.7	8 weeks
Taaffe DR	2019	Australia	Prostate cancer	54/50	104/0	IMEX:69.0 ± 6.3; Ctrl:67.5 ± 7.7	6 months
Ford KL	2024	Canada	Colorectal cancer	25/25	30/20	Exp:58 ± 9; Ctrl:57 ± 13	12 weeks
Laviano A	2020	Croatia, Italy, Slovakia, Sweden	Non-small-cell lung cancer	26/29	38/17	TMN: 64.4 ± 7.7; Ctrl: 66.0 ± 8.0	12 weeks
Uth J	2016	Denmark	Prostate cancer	29/28	57/0	Exp: 67.1 ± 7.1; Ctrl: 66.5 ± 4.9	32 weeks
Dawson et al.	2018/2021	USA	Prostate cancer	37 randomized; 32 primary analysis; 17 secondary subset	17/0	EXE: 64.0 [62.0, 72.0]; Ctrl: 65.5 [60.5, 72.5]	12 weeks
Demark-Wahnefried W	2008	USA	Breast cancer	26/29/27	0/90	CA + EX:41.9 ± 4.8; CA + EX + FVLF:42.3 ± 6.2; Ctrl:41.1 ± 5.8	6 months
Aredes MA	2019	Brazil	Cervical cancer	20/20	0/40	IG:45.14 ± 9.67; Ctrl:43.90 ± 7.88	45 days
Adams SC	2016	Canada	Breast cancer	66/64/70	0/200	Overall:48.8, range 25–78; group-specific mean ± SD NR	17 weeks
van der Werf A	2020	Netherlands	Metastatic colorectal cancer	51/54	66/39	NC:64 ± 13; Ctrl:66 ± 10	T1: mean 80 days; T2: mean 19 ± 3 weeks
D’Silva C	2025	India	Head and neck cancer	38/39	62/18	IG:54.26 ± 10.43; Ctrl:54.59 ± 11.10	7 weeks
Allen SK	2022	UK	Esophagogastric cancer	26/28	46/8	Prehab:65 ± 6; Ctrl:62 ± 9	15 weeks
Burden ST	2017	UK	Colorectal cancer	55/45	67/34	ONS:70.5 ± 11.66; Ctrl:68.9 ± 11.49	5–7 days post-operative; complications followed to 30 days
Fujimoto T	2025	Japan	Gastrointestinal cancer	29/33	17/28	Exp:80.0 ± 3.4; Ctrl:80.6 ± 5.4	At discharge; 1-year postoperative follow-up
Ritch CR	2019	USA	Bladder cancer	28/24	54/7	ONS:69, median; Ctrl:67, median; mean ± SD NR	8-week perioperative period; 30-day postoperative outcomes
Uemura S	2026	Japan	Pancreatic cancer	27/30	27/30	EI:69 (39–82); Ctrl:71 (56–84); mean ± SD NR	2 months post-treatment; survival followed for OS
Anandavadivelan P	2023	Sweden	Esophageal cancer	64/70	NR	NR	12 weeks

Detailed intervention and control regimens, including nutritional and exercise prescriptions, are provided in Supplementary Table S26.

### 3.2 Risk-of-bias assessment

Risk of bias was assessed for all 21 included randomized controlled trials. Overall, one trial was judged to be at low risk, 16 as having some concerns, and four as being at high risk (Figure 2). Figure 2 presents domain-level and overall RoB 2 judgments across all included trials.

**FIGURE 2:**
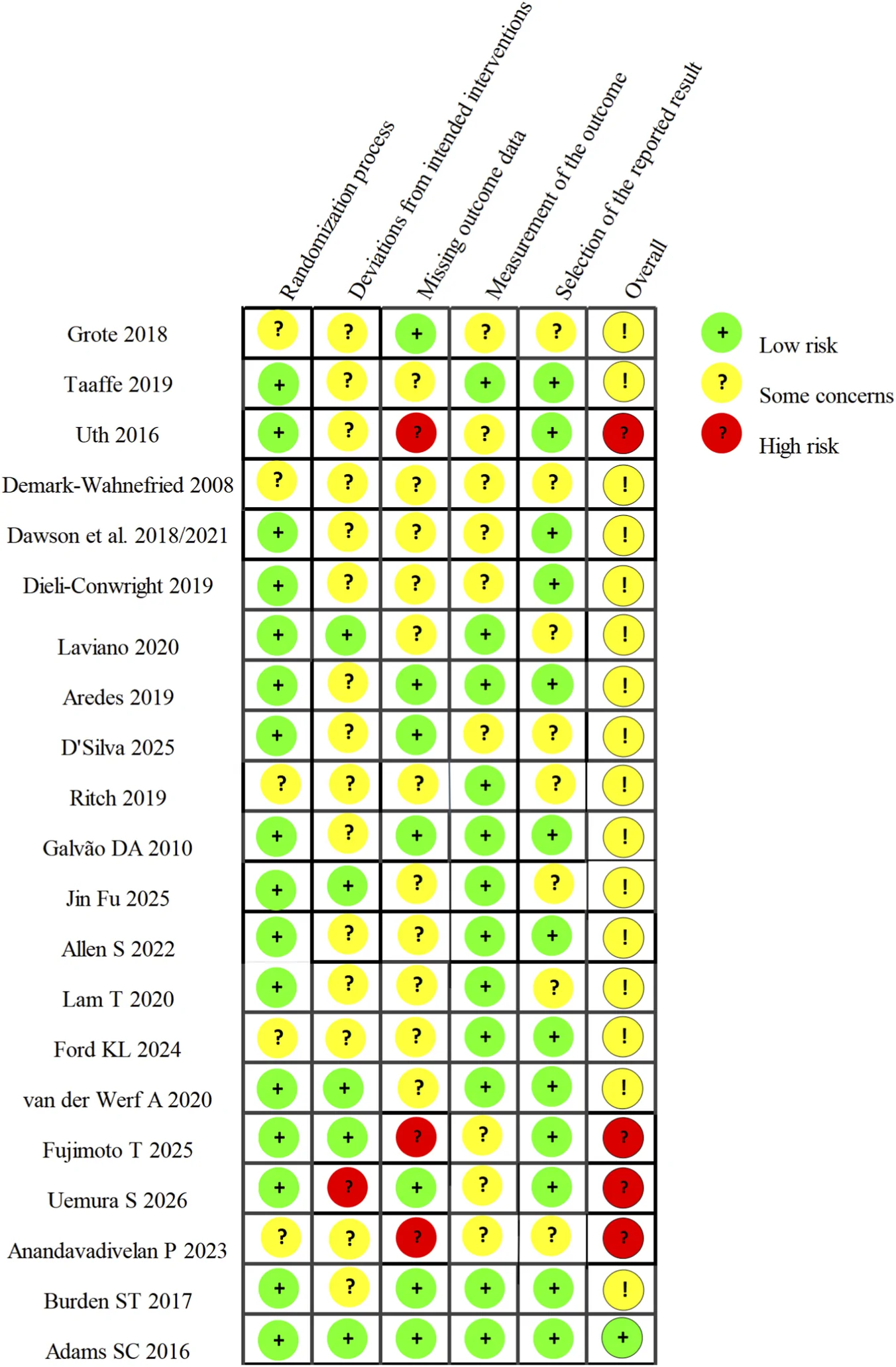
RoB 2 judgments for the 21 included randomized controlled trials. Judgments are presented for the five RoB 2 domains and overall as low risk, some concerns, or high risk.

For the randomization process, 16 trials were judged at low risk and five as having some concerns. For deviations from intended interventions, five trials were at low risk, 15 had some concerns, and one was at high risk. For missing outcome data, seven trials were at low risk, 11 had some concerns, and three were at high risk. Outcome measurement was judged at low risk in 12 trials and as having some concerns in nine; selection of the reported result was judged at low risk in 13 trials and as having some concerns in eight.

The principal concerns involved deviations from intended interventions and incomplete outcome data. Overall judgments were dominated by some concerns, while four trials were judged at high risk, primarily because of missing data or deviations from intended interventions.

### 3.3 Consistency and heterogeneity

A closed loop was present in the network for the SMI outcome; therefore, inconsistency was formally assessed. The global inconsistency test showed no statistical evidence of inconsistency (P = 0.679). Local inconsistency was further evaluated using the node-splitting method, and all evaluated comparisons had P values greater than 0.05. Loop-specific inconsistency analysis was also performed to examine agreement between direct and indirect evidence. The confidence intervals of the inconsistency factors crossed zero, suggesting no statistical evidence of inconsistency within the network (Supplementary Figure S1). Accordingly, the primary network meta-analysis for SMI was conducted using a consistency model.

For ASM, lean mass, fat mass, BMI, and body weight, the networks contained no closed loops, so inconsistency could not be estimated. The networks were sparse: the numbers of contributing trials/distinct directly observed node pairs were ASM 4/2, lean mass 5/4, fat mass 7/4, BMI 6/3, and body weight 7/3; SMI included 3 trials and 4 distinct directly observed node pairs. Edge-specific direct-comparison counts are reported in Supplementary Table S3.

Transitivity was not assumed from age alone. Control-group mean age ranged from 41.1 years in Demark-Wahnefried 2008 (breast cancer, outpatient chemotherapy) to 80.6 years in Fujimoto 2025 (gastrointestinal cancer, perioperative setting), and studies also differed in tumor site, treatment stage, baseline nutritional status, intervention intensity, follow-up, and measurement method. These differences may modify treatment effects and weaken indirect comparisons; indirectness was therefore downgraded where appropriate.

### 3.4 Muscle mass outcomes

#### 3.4.1 Appendicular skeletal muscle mass

For appendicular skeletal muscle mass (ASM), data were synthesized from four studies and three intervention nodes (Figure 3). ME versus SOC produced an imprecise estimate that crossed the null (MD -4.92 kg, 95% CI -13.46 to 3.63), as did NOI versus SOC (MD -0.80 kg, 95% CI -15.51 to 13.91) (Figure 4). These estimates do not establish benefit or harm.

**FIGURE 3:**
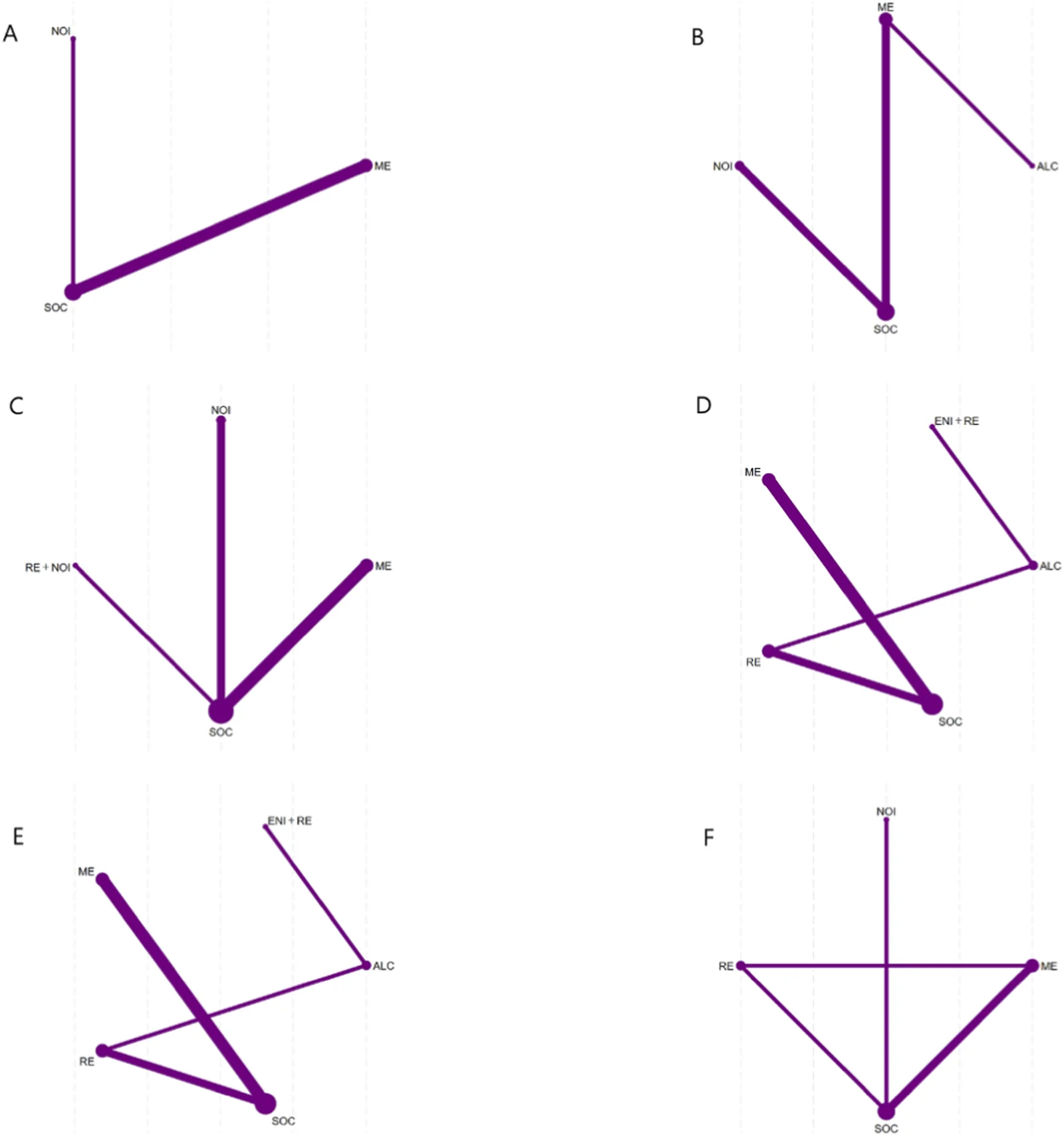
Network maps for adults with cancer: **(A)** appendicular skeletal muscle mass (ASM); **(B)** body mass index (BMI); **(C)** body weight; **(D)** lean mass; **(E)** fat mass; and **(F)** skeletal muscle index (SMI). Line thickness reflects the number of direct trial comparisons. Note: Symbols indicate certainty of evidence according to GRADE classification: * = high; + = moderate; ‡ = low; § = very low.

**FIGURE 4:**
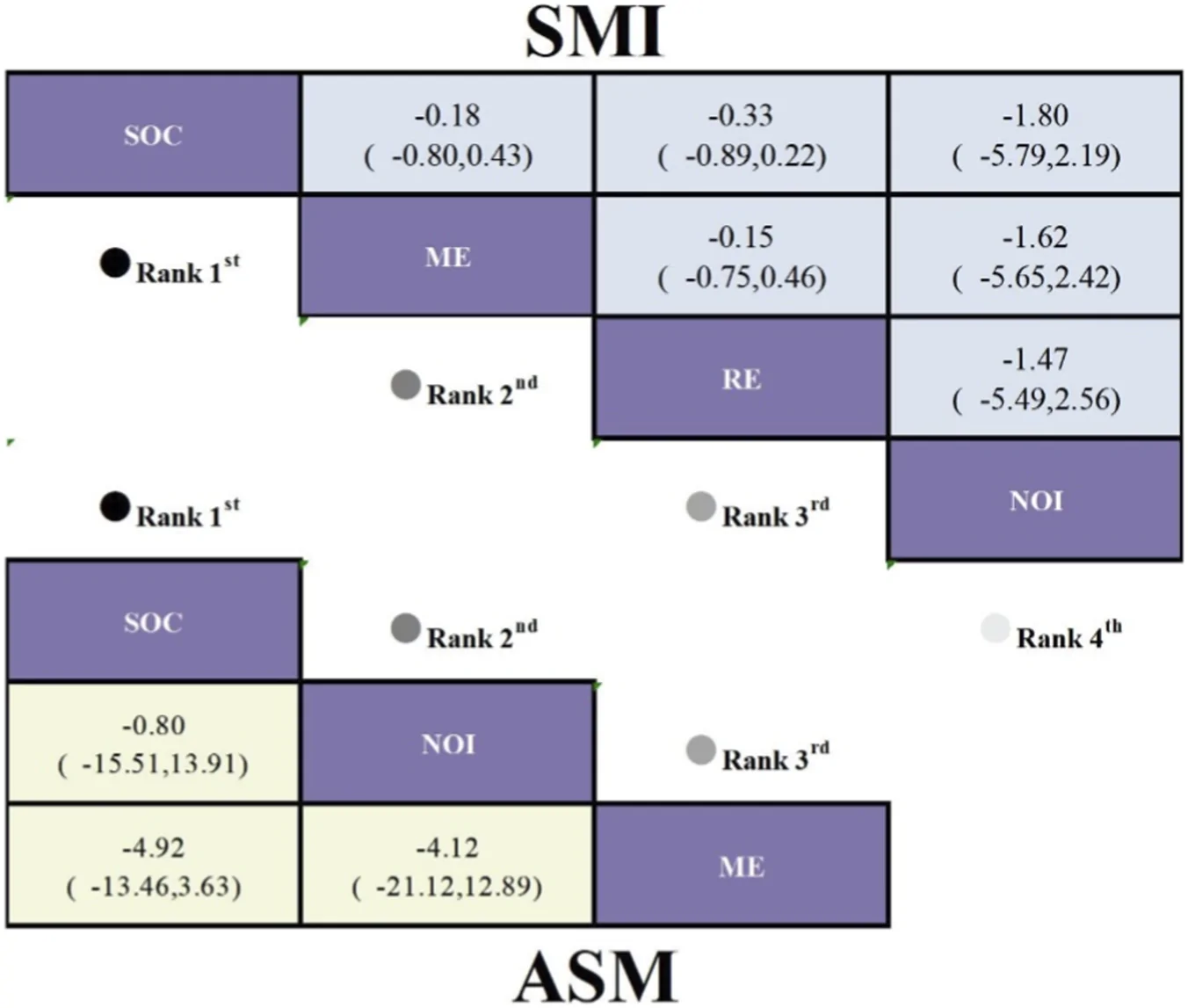
League plots of the network meta-analyses for SMI and ASM.

According to the SUCRA results, SOC had the highest SUCRA value (70.0%), followed by NOI (58.1%) and ME (22.0%), suggesting that SOC and NOI ranked relatively favorably for ASM (Supplementary Figure S2). Probability ranking showed that NOI had the highest probability of being ranked best (46.0%), followed by SOC (36.6%) and ME (8.1%). Thus, although SOC had the highest SUCRA value, NOI appeared more likely to be ranked first. The mean-rank analysis further supported this pattern: SOC had the lowest mean rank (1.6), followed by NOI (1.8) and ME (2.6), indicating that SOC and NOI had the most favorable overall rankings for the ASM outcome (Supplementary Table S9).

#### 3.4.2 Skeletal muscle index

For skeletal muscle index (SMI), data were synthesized from three studies and four intervention nodes. Estimates for ME versus SOC (MD -0.18, 95% CI -0.80 to 0.43), RE versus SOC (MD -0.33, 95% CI -0.89 to 0.22), and NOI versus SOC (MD -1.80, 95% CI -5.79 to 2.19) were imprecise and crossed the null (Figure 4).

According to the SUCRA results, SOC had the highest SUCRA value (80.8%), followed by ME (57.2%), RE (39.9%), and NOI (22.1%), suggesting that SOC and ME ranked relatively favorably for the SMI outcome (Supplementary Figure S3). Probability ranking showed that SOC had the highest probability of being ranked first (55.6%), followed by ME (20.2%), NOI (18.5%), and RE (5.7%). Thus, SOC had the most favorable ranking according to both SUCRA and the probability of being ranked first. The mean-rank analysis further supported this pattern: SOC had the lowest mean rank (1.6), followed by ME (2.3), RE (2.8), and NOI (3.3), indicating that SOC and ME had relatively favorable overall rankings for SMI (Supplementary Table S10).

#### 3.4.3 Lean mass

For lean mass, data were synthesized from five studies and five intervention nodes. ENI + RE was associated with lower lean mass than SOC (MD -6.72, 95% CI -12.43 to −1.02), but the estimate arose from a sparse network and low-certainty evidence. ALC, ME, and RE did not differ statistically from SOC (Figure 5).

**FIGURE 5:**
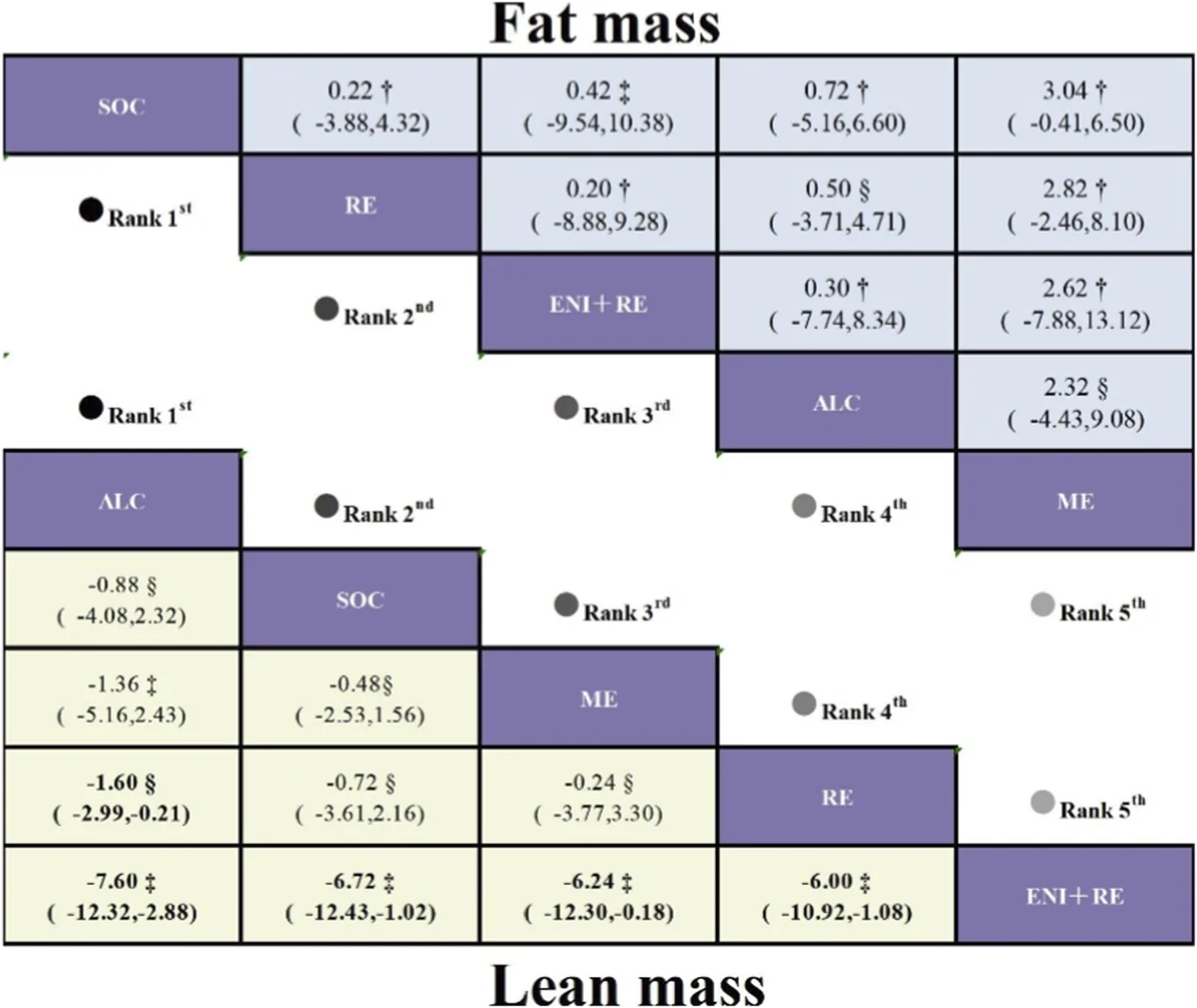
League plots of the network meta-analyses for fat mass and lean mass.

According to the SUCRA results, ALC had the highest SUCRA value (86.4%), followed by SOC (65.9%), ME (52.4%), RE (44.1%), and ENI + RE (1.2%), suggesting that ALC and SOC ranked relatively favorably for lean mass (Supplementary Figure S4). Probability ranking showed that ALC had the highest probability of being ranked first (65.3%), followed by SOC (19.8%), ME (14.5%), RE (0.4%), and ENI + RE (0.1%). Thus, ALC ranked most favorably according to both SUCRA and the probability of being ranked first. The mean-rank analysis further supported this pattern: ALC had the lowest mean rank (1.5), followed by SOC (2.4), ME (2.9), RE (3.2), and ENI + RE (5.0), indicating that ALC and SOC had relatively favorable overall rankings for lean mass (Supplementary Table S11).

### 3.5 Body weight and body composition outcomes

#### 3.5.1 Body weight

For body weight, data were synthesized from seven studies and four intervention nodes. Estimates for ME, NOI, and RE + NOI versus SOC were imprecise and crossed the null (Figure 6); therefore, the data did not establish a difference in body weight between these interventions and SOC.

**FIGURE 6:**
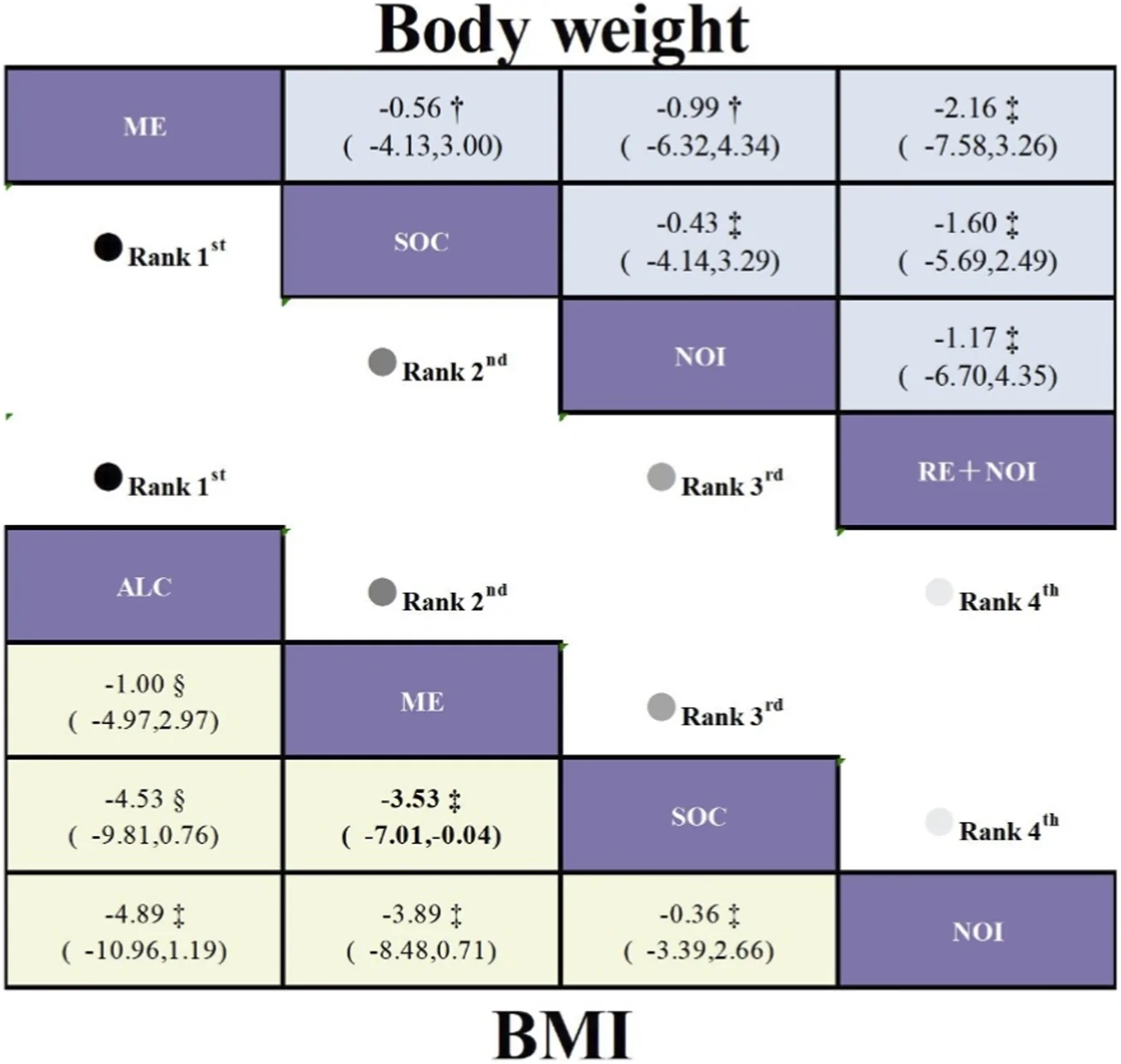
League plots of the network meta-analyses for body weight and BMI.

According to the SUCRA results, ME had the highest SUCRA value (68.4%), followed by SOC (58.1%), NOI (47.9%), and RE + NOI (25.7%), suggesting that ME and SOC ranked relatively favorably for body weight (Supplementary Figure S5). Probability ranking showed that ME had the highest probability of being ranked first (48.6%), followed by NOI (24.6%), SOC (15.9%), and RE + NOI (10.8%). Thus, ME ranked most favorably according to both SUCRA and the probability of being ranked first. The mean-rank analysis further supported this pattern: ME had the lowest mean rank (1.9), followed by SOC (2.3), NOI (2.6), and RE + NOI (3.2), indicating that ME and SOC had relatively favorable overall rankings for body weight (Supplementary Table S12).

#### 3.5.2 Body mass index

For BMI, data were synthesized from six studies and four intervention nodes. ME was associated with lower BMI than SOC (MD -3.53, 95% CI -7.01 to −0.04), whereas the estimates for ALC and NOI crossed the null (Figure 6). Given the low certainty, sparse direct evidence, and uncertain clinical meaning of BMI reduction in cancer, this finding should not be interpreted as clinical superiority.

According to the SUCRA results, ALC had the highest SUCRA value (86.4%), followed by ME (74.5%), SOC (22.2%), and NOI (16.9%), suggesting that ALC and ME ranked relatively favorably for BMI (Supplementary Figure S6). Probability ranking showed that ALC had the highest probability of being ranked first (68.6%), followed by ME (28.9%), NOI (1.9%), and SOC (0.6%). Thus, ALC ranked most favorably according to both SUCRA and the probability of being ranked first. The mean-rank analysis further supported this pattern: ALC had the lowest mean rank (1.4), followed by ME (1.8), SOC (3.3), and NOI (3.5), indicating that ALC and ME had relatively favorable overall rankings for BMI (Supplementary Table S13).

#### 3.5.3 Fat mass

For fat mass, data were synthesized from seven studies and five intervention nodes. None of the estimates comparing RE, ENI + RE, ALC, or ME with SOC excluded the null (Figure 5). The available evidence therefore did not establish a difference in fat mass between any active intervention and SOC.

Pairwise comparisons among active interventions also showed no statistically significant differences. According to the SUCRA ranking, ME had the highest SUCRA value (81.5%), with a 58.1% probability of being ranked first and a mean rank of 1.7. This was followed by ALC (SUCRA 48.7%, mean rank 3.1), ENI + RE (SUCRA 45.6%, mean rank 3.2), RE (SUCRA 40.0%, mean rank 3.4), and SOC (SUCRA 34.2%, mean rank 3.6) (Supplementary Figure S7; Supplementary Table S14).

### 3.6 Sensitivity analysis and assessment of reporting bias

Formal subgroup analyses and network meta-regression were not performed because each outcome network contained too few trials to support reliable effect-modifier analyses. Clinical heterogeneity was instead examined qualitatively across cancer type and treatment setting, exercise modality, intensity and duration, supplement type, baseline characteristics, and measurement method.

Leave-one-out sensitivity analyses were performed for networks that remained connected after study exclusion. Several estimates changed in magnitude or direction, and exclusion of the Dawson companion report disconnected the lean-mass and fat-mass networks. The sensitivity analyses therefore did not establish robust comparative effects; instead, they reinforced the fragility expected from sparse networks (Supplementary Tables S15–S18).

No outcome network met the prespecified threshold of at least 10 studies for a comparison-adjusted funnel plot. Funnel plots were therefore not generated, and no claim about the absence of publication bias was made.

### 3.7 Certainty of evidence assessment

The certainty of network estimates was assessed using GRADE/CINeMA (Supplementary Tables S19–S24). Across the six outcome networks, most comparisons were rated low certainty; no comparison was rated high certainty.

Comparisons with relatively higher certainty were generally supported by multiple direct head-to-head trials, with confidence intervals that crossed neither the line of no effect nor the prespecified MID threshold. In contrast, comparisons rated as low or very low certainty were mainly limited by sparse network structures, reliance on indirect evidence, wide confidence intervals, or potential small-study effects.

## 4 Discussion

### 4.1 Main findings

This network meta-analysis synthesized 21 randomized controlled trials evaluating eight exercise, nutrition, and combined-intervention nodes in adults with cancer. Direct head-to-head evidence was limited and most network estimates were of low certainty; the findings therefore require cautious interpretation.

For muscle mass outcomes, most estimates crossed the null and had wide confidence intervals. For lean mass, the ENI + RE estimate favored SOC, but this counterintuitive result should not be read as evidence that exercise plus nutrition reduces muscle mass. It was informed by a sparse network, heterogeneous multicomponent protocols and clinical settings, and low-certainty evidence. Differences in baseline nutritional status, cancer treatment and perioperative timing, intervention adherence, concomitant weight change, and the use of DXA, BIA, or CT may also have contributed. Residual baseline imbalance and selective availability of outcome data cannot be excluded.

For BMI, body weight, and fat mass, some interventions occupied higher relative ranks, but rank is not equivalent to efficacy. A higher SUCRA value can occur even when all pairwise estimates are imprecise or cross the null. In cancer care, a reduction in BMI or body weight may reflect unwanted loss of lean tissue rather than benefit. Rankings were therefore interpreted only with the corresponding effect estimates, confidence intervals, and certainty ratings.

The included evidence was clinically heterogeneous. Exercise interventions ranged from home-based activity and recreational football to supervised progressive resistance and multicomponent programmes. Intensity ranged from low-intensity activity or stretching controls to moderate-to-vigorous aerobic exercise and resistance exercise prescribed at approximately 60%–83% of one-repetition maximum; frequency and duration also varied, commonly from two to three sessions per week and from 7 weeks to 12 months. Nutritional interventions included dietary counselling, protein targets, whey protein or amino-acid supplementation, oral nutritional supplements, omega-3 fatty acids, and elemental diets, with substantial differences in energy content, dose, timing, and monitoring. These components were combined within broad network nodes, so the analysis cannot determine whether effects differ by exercise modality, intensity, frequency, duration, or supplement type.

A central limitation is that most intervention categories pooled clinically diverse protocols and were connected by few direct comparisons. Open-label exercise and counselling designs, incomplete reporting of allocation concealment and adherence, and attrition contributed to RoB 2 concerns. Together with tumor and treatment heterogeneity, these factors limit transitivity and the clinical interpretation of the network estimates.

Clinical context also varied materially. Trials enrolled patients with breast, prostate, head and neck, gastrointestinal, pancreatic, cervical, bladder, lung, and mixed cancers during chemotherapy, radiotherapy, androgen-deprivation therapy, perioperative care, or survivorship. Exercise tolerance, anabolic responsiveness, nutritional deficits, treatment toxicity, and expected changes in body composition may differ across these settings. The evidence base also spans 2008–2026, during which supportive care, anticancer treatment, body-composition assessment, and exercise-prescription standards evolved. These differences may modify treatment effects and weaken the exchangeability required for indirect comparisons.

Accordingly, the apparent relative rankings should be interpreted as hypotheses rather than evidence that a particular programme or supplement is optimal. The sparse outcome networks did not support reliable subgroup analyses or network meta-regression by cancer site, treatment phase, exercise dose, intervention duration, or supplement composition. Future trials should report these characteristics consistently and prespecify adequately powered effect-modifier analyses.

### 4.2 Interpretation of findings

The relatively favorable ranking of ME for BMI, body weight, and fat mass in the present study may be explained by several biological, behavioral, and clinical-care mechanisms. Biologically, multicomponent exercise typically combines aerobic training, resistance exercise, and functional training. Aerobic exercise can increase energy expenditure, improve insulin sensitivity, and promote fat oxidation, whereas resistance exercise can stimulate muscle protein synthesis, improve muscle strength, and attenuate cancer-related inflammation and disuse-related muscle atrophy (35–38). Together, these effects may partly explain the favorable ranking of ME for body weight, BMI, and fat mass (35–38). Current guidelines and reviews also suggest that exercise may help mitigate cancer cachexia- and sarcopenia-related changes by modulating muscle metabolism, inflammation, insulin sensitivity, and mitochondrial function (8, 39, 40).

From a behavioral perspective, ME may be more acceptable than single-component interventions because it integrates aerobic, resistance, flexibility, and functional training components, which may enhance patient engagement and reduce fatigue or poor adherence associated with repetitive training (41, 42). Multicomponent exercise can also be adapted to patients’ physical capacity, treatment stage, and symptom burden, potentially improving feasibility and sustainability in real-world clinical settings (35, 41, 42). In contrast, nutrition-only interventions (NOI) mainly aim to improve nutritional status by increasing energy and protein intake; however, without a concurrent exercise stimulus, they may be insufficient to fully promote muscle anabolism. Previous guidelines and reviews have similarly suggested that dietary counselling or oral nutritional supplementation may support body weight management in cancer cachexia, but evidence for muscle mass-related outcomes remains limited and inconsistent (7, 43). The absence of a consistent advantage for exercise-plus-nutrition interventions incorporating resistance exercise (ENI + RE) may reflect the small number of included studies, heterogeneity in intervention protocols, variation in nutritional dose and exercise intensity, and differences in patients’ baseline nutritional status (44).

From a clinical-care perspective, ME often involves assessment, supervision, and feedback from healthcare professionals or exercise specialists. This ongoing support may facilitate timely adjustment of exercise intensity, monitoring of adverse events, enhancement of patient self-efficacy, and maintenance of physical activity during cancer treatment (8, 35, 41). The ESPEN guideline also emphasizes that nutritional management and physical activity recommendations for patients with cancer should be based on individualized assessment and delivered by a multidisciplinary team (8).

These explanations remain hypotheses and do not establish that ME is superior to NOI or ENI + RE. The sparse networks and low certainty do not support causal conclusions about exercise modality, supplement type, or their interaction. Future trials should report intervention intensity and adherence, baseline nutritional status, treatment phase, and standardized body-composition measurements so that clinically credible subgroup analyses can be undertaken (7, 8, 35–44).

### 4.3 Implications for clinical practice and research

This review synthesized 21 randomized controlled trials across eight intervention nodes. Although ME occupied a higher relative rank for some body composition outcomes, the rankings do not establish clinical superiority because direct evidence was sparse, most estimates were uncertain, and changes in BMI or body weight are not necessarily beneficial in adults with cancer.

NOI and ENI + RE showed inconsistent estimates for ASM, SMI, and lean mass. The current evidence is therefore insufficient to recommend one intervention category over another for preserving muscle mass. Clinical decisions should instead be individualized according to treatment phase, nutritional status, functional capacity, patient goals, and the feasibility and safety of the specific protocol.

Clinical implementation should be guided by shared decision-making among patients and multidisciplinary teams, including physicians, dietitians, and rehabilitation or exercise specialists. Intervention selection should consider patient-specific goals, such as weight control, fat mass reduction, or maintenance of muscle mass; intervention duration, which commonly ranged from 8 to 16 weeks and extended to 6 months in some studies; and the patient’s expected adherence and functional capacity.

Future adequately powered head-to-head trials should stratify or prespecify analyses by cancer type and treatment setting; report exercise modality, intensity, frequency, session duration, programme duration, progression, supervision, and adherence; specify supplement composition, energy and protein content, dose, timing, and compliance; and use standardized DXA- or CT-based outcomes where appropriate. Such reporting is needed before reliable subgroup or effect-modifier analyses can identify which components benefit which patients.

### 4.4 Comparison with previous studies

Previous studies have examined the effects of exercise and nutritional interventions on sarcopenia-related outcomes in patients with cancer. Liu et al. conducted a network meta-analysis including 10 RCTs and 503 patients with cancer, mainly comparing aerobic exercise, resistance exercise, combined aerobic and resistance exercise, and stretching. Their results showed that combined aerobic and resistance exercise ranked highest for reducing fat mass, whereas resistance exercise showed advantages in improving lean mass and leg extension function (10). These findings are partly consistent with the present study, in which multicomponent exercise (ME) ranked relatively favorably for body composition-related outcomes, including fat mass, BMI, and body weight.

Several important differences should be noted. Liu et al. included only exercise interventions and did not systematically compare nutritional interventions or combined exercise–nutrition strategies. In contrast, the present study included multiple intervention nodes, including SOC, ALC, RE, ME, NOI, ENI, RE + NOI, and ENI + RE, allowing a more comprehensive evaluation of the relative effects of exercise, nutritional, and combined interventions. In addition, Liu et al. mainly focused on lean mass, fat mass, and leg extension function, whereas the present study further included ASM, SMI, BMI, and body weight, thereby providing a broader assessment of muscle mass and body composition changes.

Jang et al. conducted a systematic review and meta-analysis including six studies and reported a non-significant trend toward increased SMI after intervention, while lean body mass showed no significant change (6). The authors suggested that resistance exercise or combined exercise–nutrition interventions may help maintain or increase muscle mass. These findings are broadly consistent with the uncertainty observed in our ASM and SMI results, in which several interventions showed favorable trends but lacked stable statistical evidence. Unlike the present study, Jang et al. mainly included patients receiving chemotherapy and used a conventional pairwise meta-analysis, which could not simultaneously compare and rank multiple interventions. By contrast, the present study used network meta-analysis to synthesize direct and indirect evidence, compare the relative effects of different exercise- and nutrition-related interventions, and further assess the certainty of evidence using GRADE/CINeMA.

Differences from earlier reviews may reflect intervention classification, comparator definitions, outcome measurement, and clinical setting. Muscle mass was assessed using DXA, CT, and BIA, which quantify different tissue compartments and are not directly interchangeable. BIA is influenced by hydration and peri-treatment fluid shifts; CT is often limited to a specific anatomical level; and DXA estimates whole-body or regional lean soft tissue rather than muscle directly. Pooling across these modalities may increase heterogeneity and obscure small treatment effects.

SUCRA indicates relative ordering within a network; it does not establish that an intervention is effective, clinically important, or superior. Rankings are especially unstable when networks are sparse and estimates are imprecise. Accordingly, we interpreted SUCRA only with effect size, 95% CI, directness, and certainty. A high rank accompanied by a CI crossing the null was treated as insufficient evidence rather than as proof of benefit.

### 4.5 Strengths and limitations

This study followed a preregistered protocol and PRISMA-NMA guidance, used random-effects network models, assessed inconsistency where closed loops existed, performed leave-one-out sensitivity analyses, and evaluated certainty using CINeMA/GRADE. Detailed node definitions separated exercise, nutrition, and combined strategies. However, the classification itself remains clinically challenging because intervention components can overlap, and several nodes were supported by only a small number of trials.

Compared with previous studies, this review provides a broader and more comprehensive synthesis. Liu et al. included 10 RCTs with 503 patients and focused on aerobic exercise, resistance exercise, combined exercise, and stretching, without systematically comparing nutritional or combined exercise–nutrition interventions. Jang et al. included six studies and mainly focused on patients receiving chemotherapy, but their pairwise meta-analysis could not compare the relative rankings of multiple interventions. In contrast, the present study included multiple intervention nodes, including SOC, ALC, RE, ME, NOI, ENI, RE + NOI, and ENI + RE, and expanded the outcomes to include body composition indicators such as ASM, SMI, BMI, and body weight. This study therefore provides a more systematic comparative basis for evaluating the relative effects of exercise, nutritional, and combined interventions.

Several limitations require emphasis. Direct evidence was sparse and most networks had no closed loops, so many estimates relied on indirect comparisons and inconsistency could not be tested. Transitivity may be compromised by differences in cancer type, treatment stage, age, baseline nutritional status, exercise dose and supervision, supplement composition, intervention duration, and control care. Body composition was measured with DXA, BIA, or CT, which are not interchangeable and may respond differently to hydration and treatment-related changes. The small number of studies per outcome precluded credible subgroup analysis or meta-regression. Risk-of-bias concerns and wide confidence intervals further reduced certainty.

These limitations mean that effect estimates and rankings should not be translated into definitive clinical recommendations. The apparent reduction in lean mass with ENI + RE may reflect sparse evidence, clinical and measurement heterogeneity, or baseline imbalance rather than a harmful effect of combined treatment. The findings should therefore be used to guide future comparative research, not to select a single preferred intervention.

### 4.6 Conclusion

The available evidence does not identify a consistently superior exercise, nutritional, or combined intervention for muscle mass or body composition outcomes in adults with cancer. ME was associated with lower BMI than SOC, but the clinical meaning is uncertain and the result should not be interpreted as proof of benefit. The lean-mass estimate for ENI + RE was also low certainty and should not be interpreted as evidence of harm.

Sparse direct evidence, heterogeneous cancer and treatment settings, varied exercise and nutrition protocols, different measurement methods, and low certainty limit the conclusions. Standardized, adequately powered head-to-head trials are needed before comparative rankings can guide practice.

## Data Availability

The original contributions presented in the study are included in the article/Supplementary Material, further inquiries can be directed to the corresponding author.

## References

[1] Westerink NL Nuver J Lefrandt JD Vrieling AH Gietema JA Walenkamp AM . Cancer treatment induced metabolic syndrome: improving outcome with lifestyle. Crit Rev Oncol Hematol (2016) 108:128–36. 10.1016/j.critrevonc.2016.10.011 27931830

[2] Bray F Laversanne M Sung H Ferlay J Siegel RL Soerjomataram I Global cancer statistics 2022: GLOBOCAN estimates of incidence and mortality worldwide for 36 cancers in 185 countries. CA Cancer J Clin (2024) 74:229–63. 10.3322/caac.21834 38572751

[3] Cruz-Jentoft AJ Bahat G Bauer J Boirie Y Bruyère O Cederholm T Sarcopenia: revised European consensus on definition and diagnosis. Age Ageing (2019) 48:16–31. 10.1093/ageing/afy169 30312372 PMC6322506

[4] Surov A Wienke A . Prevalence of sarcopenia in patients with solid tumors: a meta-analysis based on 81, 814 patients. JPEN J Parenter Enteral Nutr (2022) 46:1761–8. 10.1002/jpen.2415 35633306

[5] Davis MP Panikkar R . Sarcopenia associated with chemotherapy and targeted agents for cancer therapy. Ann Palliat Med (2019) 8:86–101. 10.21037/apm.2018.08.02 30525762

[6] Jang MK Park C Tussing-Humphreys L Fernhall B Phillips S Doorenbos AZ . The effectiveness of sarcopenia interventions for cancer patients receiving chemotherapy: a systematic review and meta-analysis. Cancer Nurs (2023) 46:E81–e90. 10.1097/ncc.0000000000000957 34054070 PMC8627517

[7] Roeland EJ Bohlke K Baracos VE Bruera E Del Fabbro E Dixon S Management of cancer cachexia: ASCO guideline. J Clin Oncol (2020) 38:2438–53. 10.1200/JCO.20.00611 32432946

[8] Muscaritoli M Arends J Bachmann P Baracos V Barthelemy N Bertz H ESPEN practical guideline: Clinical nutrition in cancer. Clin Nutr (2021) 40:2898–913. 10.1016/j.clnu.2021.02.005 33946039

[9] Zhao Y Ying L Gao X Tang L Zhang Y Pan W Sarcopenia interventions targeted at improving muscle health in adults with cancer: a systematic review and meta-analysis. Front Nutr (2025) 12:1671720. 10.3389/fnut.2025.1671720 41561181 PMC12812668

[10] Liu R Gao XY Wang L . Network meta-analysis of the intervention effects of different exercise measures on sarcopenia in cancer patients. BMC Public Health (2024) 24:1281. 10.1186/s12889-024-18493-y 38730397 PMC11083843

[11] Hutton B Salanti G Caldwell DM Chaimani A Schmid CH Cameron C The PRISMA extension statement for reporting of systematic reviews incorporating network meta-analyses of health care interventions: checklist and explanations. Ann Intern Med (2015) 162:777–84. 10.7326/M14-2385 26030634

[12] Sterne JAC Savovic J Page MJ Elbers RG Blencowe NS Boutron I RoB 2: a revised tool for assessing risk of bias in randomised trials. BMJ (2019) 366:l4898. 10.1136/bmj.l4898 31462531

[13] Fu J Yu K Zhang Y Bao Y Li S . Effects of FSMP on nutrition status and sarcopenia among nutritional risk cancer patients: a randomized, double-blind, placebo-controlled study. Asia Pac J Clin Nutr (2025) 34:566–76. 10.6133/apjcn.202508_34(4).0008 40738724 PMC12310436

[14] Galvão DA Taaffe DR Spry N Joseph D Newton RU . Combined resistance and aerobic exercise program reverses muscle loss in men undergoing androgen suppression therapy for prostate cancer without bone metastases: a randomized controlled trial. J Clin Oncol (2010) 28:340–7. 10.1200/JCO.2009.23.2488 19949016

[15] Lam T Cheema B Hayden A Lord SR Gurney H Gounden S Androgen deprivation in prostate cancer: benefits of home-based resistance training. Sports Med Open (2020) 6:59. 10.1186/s40798-020-00262-1 33315154 PMC7736381

[16] Dieli-Conwright CM Sweeney FC Courneya KS Tripathy D Sami N Lee K Hispanic ethnicity as a moderator of the effects of aerobic and resistance exercise in survivors of breast cancer. Cancer (2019) 125:910–20. 10.1002/cncr.31879 30500981 PMC7164690

[17] Grote M Maihöfer C Weigl M Davies-Knorr P Belka C . Progressive resistance training in cachectic head and neck cancer patients undergoing radiotherapy: a randomized controlled pilot feasibility trial. Radiat Oncol (2018) 13:215. 10.1186/s13014-018-1158-0 30400971 PMC6219249

[18] Taaffe DR Galvão DA Spry N Joseph D Chambers SK Gardiner RA Immediate *versus* delayed exercise in men initiating androgen deprivation: effects on bone density and soft tissue composition. BJU Int (2019) 123:261–9. 10.1111/bju.14505 30239116 PMC6635752

[19] Ford KL Sawyer MB Ghosh S Trottier CF Disi IR Easaw J Feasibility of two levels of protein intake in patients with colorectal cancer: findings from the protein recommendation to increase muscle (PRIMe) randomized controlled pilot trial. ESMO Open (2024) 9:103604. 10.1016/j.esmoop.2024.103604 38935990 PMC11260369

[20] Laviano A Calder PC Schols A Lonnqvist F Bech M Muscaritoli M . Safety and tolerability of targeted medical nutrition for cachexia in non-small-cell lung cancer: a randomized, double-blind, controlled pilot trial. Nutr Cancer (2020) 72:439–50. 10.1080/01635581.2019.1634746 31290697

[21] Uth J Hornstrup T Christensen JF Christensen KB Jørgensen NR Schmidt JF Efficacy of recreational football on bone health, body composition, and physical functioning in men with prostate cancer undergoing androgen deprivation therapy: 32-Week follow-up of the FC prostate randomised controlled trial. Osteoporos Int (2016) 27:1507–18. 10.1007/s00198-015-3399-0 26572756

[22] Dawson JK Dorff TB Schroeder ET Lane CJ Gross ME Dieli-Conwright CM . Impact of resistance training on body composition and metabolic syndrome variables during androgen deprivation therapy for prostate cancer: a pilot randomized controlled trial. BMC Cancer (2018) 18:368. 10.1186/s12885-018-4306-9 29614993 PMC5883585

[23] Dawson JK Dorff TB Tuzon C Rice JC Schroeder ET Lane CJ Effect of periodized resistance training on skeletal muscle during androgen deprivation therapy for prostate cancer: a pilot randomized trial. Integr Cancer Ther (2021) 20:15347354211035442. 10.1177/15347354211035442 34301165 PMC8312192

[24] Demark-Wahnefried W Case LD Blackwell K Marcom PK Kraus W Aziz N Results of a diet/exercise feasibility trial to prevent adverse body composition change in breast cancer patients on adjuvant chemotherapy. Clin Breast Cancer (2008) 8:70–9. 10.3816/CBC.2008.n.005 18501061

[25] Aredes MA da Camara AO de Paula NS Fraga KYD do Carmo M Chaves GV . Efficacy of ω-3 supplementation on nutritional status, skeletal muscle, and chemoradiotherapy toxicity in cervical cancer patients: a randomized, triple-blind, clinical trial conducted in a middle-income country. Nutr Burbank, Los Angeles County, Calif (2019) 67-68:110528. 10.1016/j.nut.2019.06.009 31445316

[26] Adams SC Segal RJ McKenzie DC Vallerand JR Morielli AR Mackey JR Impact of resistance and aerobic exercise on sarcopenia and dynapenia in breast cancer patients receiving adjuvant chemotherapy: a multicenter randomized controlled trial. Breast Cancer Res Treat (2016) 158:497–507. 10.1007/s10549-016-3900-2 27394134

[27] van der Werf A Langius JAE Beeker A Ten Tije AJ Vulink AJ Haringhuizen A The effect of nutritional counseling on muscle mass and treatment outcome in patients with metastatic colorectal cancer undergoing chemotherapy: a randomized controlled trial. Clin Nutr (2020) 39:3005–13. 10.1016/j.clnu.2020.01.009 32037284

[28] D'Silva C Shetty V Fernandes D Jean-Pierre B Kumari NS Srivastava S Effect of structured exercise-based rehabilitation on sarcopenia and quality of life among head and neck cancer patients undergoing chemo-radiotherapy: a randomized controlled trial. Asian Pac J Cancer Prev (2025) 26:1653–60. 10.31557/APJCP.2025.26.5.1653 40439377 PMC12290174

[29] Allen SK Brown V White D King D Hunt J Wainwright J Multimodal prehabilitation during neoadjuvant therapy prior to esophagogastric cancer resection: effect on cardiopulmonary exercise test performance, muscle mass and quality of Life-A pilot randomized clinical trial. Ann Surg Oncol (2022) 29:1839–50. 10.1245/s10434-021-11002-0 34725764

[30] Burden ST Gibson DJ Lal S Hill J Pilling M Soop M Pre-operative oral nutritional supplementation with dietary advice *versus* dietary advice alone in weight-losing patients with colorectal cancer: a single-blind randomized controlled trial. J Cachexia Sarcopenia Muscle (2017) 8:437–46. 10.1002/jcsm.12170 28052576 PMC5476846

[31] Fujimoto T Akasaka H Yasunobe Y Yoshida S Onishi Y Minami T Pre-operative resistance training and amino acid supplementation in frail patients with gastrointestinal cancer: a randomized clinical trial. Geriatr Gerontol Int (2025) 25:1765–73. 10.1111/ggi.70226 41108598 PMC12719127

[32] Ritch CR Cookson MS Clark PE Chang SS Fakhoury K Ralls V Perioperative oral nutrition supplementation reduces prevalence of sarcopenia following radical cystectomy: results of a prospective randomized controlled trial. J Urol (2019) 201:470–7. 10.1016/j.juro.2018.10.010 30359680

[33] Uemura S Iwashita T Koizumi T Ohashi Y Iwata S Maruta A Early elemental diet supplementation during chemotherapy improves treatment response and survival in advanced pancreatic cancer: a single-center randomized controlled trial. Clin Nutr ESPEN (2026) 73:103106. 10.1016/j.clnesp.2026.103106 41794391

[34] Anandavadivelan P Malberg K Vikstrom K Nielsen S Holdar U Johar A Home-based physical activity after treatment for esophageal cancer-A randomized controlled trial. Cancer Med (2023) 12:3477–87. 10.1002/cam4.5131 36812121 PMC9939163

[35] Campbell KL Winters-Stone KM Wiskemann J May AM Schwartz AL Courneya KS Exercise guidelines for cancer survivors: consensus statement from international multidisciplinary roundtable. Med Sci Sports Exerc (2019) 51:2375–90. 10.1249/MSS.0000000000002116 31626055 PMC8576825

[36] Montalvo RN Hardee JP VanderVeen BN Carson JA . Resistance exercise's ability to reverse cancer-induced anabolic resistance. Exerc Sport Sci Rev (2018) 46:247–53. 10.1249/JES.0000000000000159 30001273 PMC6138526

[37] Halle JL Counts BR Carson JA . Exercise as a therapy for cancer-induced muscle wasting. Sports Med Health Sci (2020) 2:186–94. 10.1016/j.smhs.2020.11.004 35782998 PMC9219331

[38] Padilha CS Cella PS Chimin P Voltarelli FA Marinello PC Testa MTJ Resistance training's ability to prevent cancer-induced muscle atrophy extends anabolic stimulus. Med Sci Sports Exerc (2021) 53:1572–82. 10.1249/mss.0000000000002624 33731662

[39] Arends J Strasser F Gonella S Solheim TS Madeddu C Ravasco P Cancer cachexia in adult patients: ESMO clinical practice guidelines(☆). ESMO Open (2021) 6:100092. 10.1016/j.esmoop.2021.100092 34144781 PMC8233663

[40] Tamayo-Torres E Garrido A de Cabo R Carretero J Gómez-Cabrera MC . Molecular mechanisms of cancer cachexia. Role of exercise training. Mol Aspects Med (2024) 99:101293. 10.1016/j.mam.2024.101293 39059039

[41] Kampshoff CS Jansen F van Mechelen W May AM Brug J Chinapaw MJ Determinants of exercise adherence and maintenance among cancer survivors: a systematic review. Int J Behav Nutr Phys Act (2014) 11:80. 10.1186/1479-5868-11-80 24989069 PMC4096543

[42] Ormel HL van der Schoot GGF Sluiter WJ Jalving M Gietema JA Walenkamp AME . Predictors of adherence to exercise interventions during and after cancer treatment: a systematic review. Psychooncology (2018) 27:713–24. 10.1002/pon.4612 29247584 PMC5887924

[43] Prado CM Purcell SA Laviano A . Nutrition interventions to treat low muscle mass in cancer. J Cachexia Sarcopenia Muscle (2020) 11:366–80. 10.1002/jcsm.12525 31916411 PMC7113510

[44] Okamura M Shirado K Shirai N Yagi T Inoue T Ogawa M Combined nutritional and exercise interventions for cachexia in chronic diseases: a systematic review and meta-analysis limited to cancer cachexia. Prog Rehabil Med (2026) 11:20260012. 10.2490/prm.20260012 41884877 PMC13010123

