# Supplementary material for: Comparative effects of exercise and nutritional interventions on muscle mass and body composition in adults with cancer: a systematic review and network meta-analysis of randomized controlled trials
Source: Oncol Rev. 2026 Oct 29;20:1926991. doi: 10.3389/or.2026.1926991 (PMC13755329; doi:10.3389/or.2026.1926991)
Supplement: Supplementary file 1 [file Supplementaryfile1.pdf]

Supplementary Material

Page	
Table S2	Literature search strategy
Table S3	Direct evidence and network geometry by outcome
Table S4	Node-splitting analysis for SMI
Figures S1-S7	Loop inconsistency and rank probability plots
Tables S9-S14	SUCRA ranking tables
Tables S15-S18	Leave-one-out sensitivity analyses
Tables S19-S24	GRADE/CINeMA certainty assessments
Table S25	Clinical characteristics of control groups by outcome network
Table S26	Detailed intervention and control regimens

Table S2.Literature Search Strategy	
PubMed	#1 "Sarcopenia"[Mesh] 1463<br>#2 (("Sarcopenia"[Mesh]) OR (Sarcopenias[Title/Abstract])) OR (Sarcopenia[Title/Abstract]) 26870<br>#3 "Neoplasms"[Mesh] 4222166<br>#4 ((((((((((((("Neoplasms"[Mesh]) OR (Neoplasms[Title/Abstract])) OR (Tumors[Title/Abstract])) OR (Neoplasia[Title/Abstract])) OR (Neoplasias[Title/Abstract])) OR (Neoplasm[Title/Abstract])) OR (Tumor[Title/Abstract])) OR (Cancer[Title/Abstract])) OR (Cancers[Title/Abstract])) OR ("Malignant Neoplasm"[Title/Abstract])) OR (Malignancy[Title/Abstract])) OR (Malignancies[Title/Abstract])) OR ("Malignant Neoplasms"[Title/Abstract])) OR ("Neoplasm, Malignant"[Title/Abstract])) OR ("Neoplasms, Malignant"[Title/Abstract])) 5,540,052<br>#5 #2 and #4 4876<br>#6 "Sports"[Mesh] 240,229<br>#7 ((((((((((((((((((((((("Sports"[Mesh]) OR (Sport[Title/Abstract])) OR (Athletics[Title/Abstract])) OR (Athletic[Title/Abstract])) OR (Exercise[Title/Abstract])) OR ("Activities, Physical"[Title/Abstract])) OR ("Activity, Physical"[Title/Abstract])) OR ("Physical Activities"[Title/Abstract])) OR ("Exercise, Physical"[Title/Abstract])) OR ("Exercises, Physical"[Title/Abstract])) OR ("Physical Exercise"[Title/Abstract])) OR ("Physical Exercises"[Title/Abstract])) OR ("Acute Exercise"[Title/Abstract])) OR ("Exercises, Acute"[Titl

	e/Abstract])) OR ("Exercise, Isometric"[Title/Abstract])) OR ("Exercises, Isometric"[Title/Abstract])) OR ("Isometric Exercises"[Title/Abstract])) OR ("Isometric Exercise"[Title/Abstract])) OR ("Exercise, Aerobic"[Title/Abstract])) OR ("Aerobic Exercise"[Title/Abstract])) OR ("Aerobic Exercises"[Title/Abstract])) OR ("Exercises, Aerobic"[Title/Abstract])) OR ("Exercise Training"[Title/Abstract])) OR ("Exercise Trainings"[Title/Abstract])) OR ("Training, Exercise"[Title/Abstract])) OR ("Trainings, Exercise"[Title/Abstract]) 608,775<br>#8 "Nutrition Therapy"[Mesh] 122,666<br>#9 (((("Nutrition Therapy"[Mesh]) OR ("Therapy, Nutrition"[Title/Abstract])) OR ("Medical Nutrition Therapy"[Title/Abstract])) OR ("Nutrition Therapy, Medical"[Title/Abstract])) OR ("Therapy, Medical Nutrition"[Title/Abstract]) 123,426<br>#10 "Diet"[Mesh] 366,080<br>#11 ((((((((("Diet"[Mesh]) OR (Diets[Title/Abstract])) OR ("Dietary Pattern"[Title/Abstract])) OR ("Dietary Patterns"[Title/Abstract])) OR ("Pattern, Dietary"[Title/Abstract])) OR ("Patterns, Dietary"[Title/Abstract])) OR ("Food Patterns"[Title/Abstract])) OR ("Food Pattern"[Title/Abstract])) OR ("Pattern, Food"[Title/Abstract])) OR ("Patterns, Food"[Title/Abstract]) 444,704<br>#12 #7 or #9 or #11 1,032,982<br>#13 #5 and #12 167<br>#14 ("Randomized Controlled Trial"[Publication Type]) OR ("Controlled Clinical Trial"[Publication Type]) OR (random*[Title/Abstract]) OR (randomized[Title/Abstract]) OR (randomised[Title/Abstract]) OR (randomly[Title/Abstract]) OR (placebo*[Title/Abstract]) OR (trial[Title/Abstract]) OR ("clinical trial"[Title/Abstract]) OR ("controlled clinical trial"[Title/Abstract]) OR (RCT[Title/Abstract])<br>#15 #13 and #14 139
Web of Science	1: TS=(Sarcopenia or Sarcopenias) 32349<br>2: TS=(Tumors or Neoplasia or Neoplasias or Neoplasm or Tumor or Cancer or Cancers or "Malignant Neoplasm" or Malignancy or Malignancies or "Malignant Neoplasms" or "Neoplasm, Malignant" or "Neoplasms, Malignant" or "Benign Neoplasms" or "Neoplasms, Benign" or "Neoplasm, Benign" or "Benign Neoplasm") 4157199<br>3: #1 and #2 6382<br>4: TS=(Sport or Athletics or Athletic or Exercise or "Activities, Physical" or "Activity, Physical" or "Physical Activities" or "Exercise, Physical" or "Exercises, Physical" or "Physical Exercise" or "Physical Exercises" or "Acute Exercise" or "Exercises, Acute" or "Exercise, Isometric" or "Exercises, Isometric" or "Isometric Exercises" or "Isometric Exercise" or "Exercise, Aerobic" or "Aerobic Exercise" or "Aerobic Exercises" or "Exercises, Aerobic" or "Exercise Training" or "Exercise Trainings" or "Training, Exercise" or "Trainings, Exercise") 625291<br>5: TS=(Diets or "Dietary Pattern" or "Dietary Patterns" or "Pattern, Dietary" or "Patterns, Dietary" or "Food Patterns" or "Food Pattern" or "Pattern, Food" or "Patterns, Food" or "Therapy, Nutrition" or "Medical Nutrition " or "Therapy Nutrition" or "Therapy, Medical" or “Therapy, Medical Nutrition”) 568342<br>6: #4 or #5 1162463<br>7: TS=(random* OR randomi* OR randomly<br>OR placebo* OR sham<br>OR trial OR "clinical trial" OR "controlled clinical trial"<br>OR "double blind" OR "single blind" OR "triple blind"<br>OR crossover OR "cross over" OR "parallel group"<br>OR cluster random*

	OR RCT) 3628512<br>8: #3 and #6 and #7 294
Cochrane	ID Search Hits<br>#1 MeSH descriptor: [Sarcopenia] explode all trees 1138<br>#2 Sarcopenias or #1 1138<br>#3 MeSH descriptor: [Neoplasms] explode all trees 131480<br>#4 Neoplasms or Benign or "Benign Neoplasm" or "Benign Neoplasms" or Neoplasm or Benign or Malignancy or "Neoplasm, Malignant" or "Malignant Neoplasm" or "Malignant Neoplasms" or "Neoplasms,Malignant" or Cancers or Malignancies or Cancer or Neoplasia or Neoplasm or Tumor or Tumors or Neoplasias or #3 326774<br>#5 #2 and #4 131<br>#6 MeSH descriptor: [Exercise] explode all trees 45731<br>#7 Athletics or Athletic or Sport or Exercise or "Activities, Physical" or "Activity, Physical" or "Physical Activities" or "Exercise, Physical" or "Exercises, Physical" or "Physical Exercise" or "Physical Exercises" or "Acute Exercise" or "Exercises, Acute" or "Exercise, Isometric" or "Exercises, Isometric" or "Isometric Exercises" or "Isometric Exercise" or "Exercise, Aerobic" or "Aerobic Exercise" or "Aerobic Exercises" or "Exercises, Aerobic" or "Exercise Training" or "Exercise Trainings" or "Training, Exercise" or "Trainings, Exercise" or #6 173973<br>#8 MeSH descriptor: [Nutrition Therapy] explode all trees 13272<br>#9 "Therapy, Medical Nutrition" or "Medical Nutrition Therapy" or 'Nutrition Therapy, Medical' or "Therapy, Nutrition" or Diets or "Dietary Pattern" or "Dietary Patterns" or "Pattern, Dietary" or "Patterns, Dietary" or "Food Patterns" or "Food Pattern" or "Pattern, Food" or "Patterns, Food" or #8 30105<br>#10 #7 or #9 199627<br>#11 #5 and #10 in Trials 69
Embase	#1 'exercise'/exp OR 'biometric exercise' OR 'effort' OR 'exercise capacity' OR 'exercise performance' OR 'exercise training' OR 'exertion' OR 'fitness training' OR 'fitness workout' OR 'physical conditioning, human' OR 'physical effort' OR 'physical exercise' OR 'physical exertion' OR 'physical work-out' OR 'physical workout' OR 'exercise' OR 'sport'/exp OR 'competitive gymnastics' OR 'competitive sport' OR 'sports' OR 'sport' 1469025<br>#2 'diet'/exp OR 'diet influence' OR 'diet regimen' OR 'diet surveys' OR 'dietary effect' OR 'dietary influence' OR 'dietary regimen' OR 'dietary survey' OR 'dietary surveys' OR 'dieting' OR 'diet' OR 'nutrition'/exp OR 'diet, food, and nutrition' OR 'nutrition council' OR 'nutrition phenomena' OR 'nutrition physiology' OR 'nutrition processes' OR 'nutrition research' OR 'nutrition research center' OR 'nutrition study' OR 'nutrition survey' OR 'nutrition surveys' OR 'nutritional physiological phenomena' OR 'nutritional physiology' OR 'nutritive solution' OR 'sports nutritional physiological phenomena' OR 'nutrition' 3914673<br>#3 #1 OR #2 5141869<br>#4 'sarcopenia'/exp OR 'age-related muscle atrophy' OR 'age-related muscle decline' OR 'age-related muscular decline' OR 'age-related muscular degeneration' OR 'ageing-related muscle atrophy' OR 'geriatric muscular atrophy' OR 'sarcopenia' 47313<br>#5 'neoplasm'/exp OR 'acral tumor' OR 'acral tumour' OR 'neoplasia' OR 'neoplasms' OR 'neoplastic disease' OR 'neoplastic entity' OR 'neoplastic mass' OR 'tumor' OR 'tumoral entity' OR 'tumoral mass' OR 'tumorous entity' OR 'tumorous mass' OR 'tumors' OR 'tumour' OR 'tumoural entity' OR 'tumoural mass' OR 'tumourous entity' OR 'tumourous mass' OR 'tumours' OR 'neoplasm' 8138190

#6	#4 AND #5	10627
#7	#6 AND 'randomized controlled trial'/de	411
#8	#3 AND #6 AND #7	316

Supplementary Table S3. Direct evidence and network geometry by outcome

Outcome	Contributing trials	Direct trial comparisons by node pair	Distinct direct node pairs
ASM	4	SOC-ME: 3; SOC-NOI: 1	2
SMI	3	SOC-ME: 2; SOC-NOI: 1; SOC-RE: 1; RE-ME: 1	4
Lean mass	5	SOC-ME: 2; SOC-RE: 1; RE-ALC: 1; ALC-ENI+RE: 1	4
Body weight	7	SOC-ME: 4; SOC-NOI: 2; SOC-RE+NOI: 1	3
BMI	6	SOC-ME: 3; SOC-NOI: 2; ME-ALC: 1	3
Fat mass	7	SOC-ME: 3; SOC-RE: 2; RE-ALC: 1; ALC-ENI+RE: 1	4

Note: Counts describe the final network geometry used in the analyses; multi-arm trials may contribute more than one direct node pair.

Supplementary Table S4. Node-splitting analysis for the SMI in adults with cancer

Side	Coef.	Std. Err.	Coef.	Std. Err.	Coef.	Std. Err.	P>z	tau
A C	0.1500002	0.3094996	-7.469901	18.39784	7.619902	18.40143	0.679	0.0007669
A D	-0.1844244	0.313326	-0.9696902	1345.823	0.7852658	1345.823	1	0.0000222
B C	-1.8	2.033551	0.2112462	4690.068	-1.588754	4690.068	1	0.0000055
B D	-0.3299995	0.2830338	-7.949901	18.39932	7.619902	18.40163	0.679	0.0058751

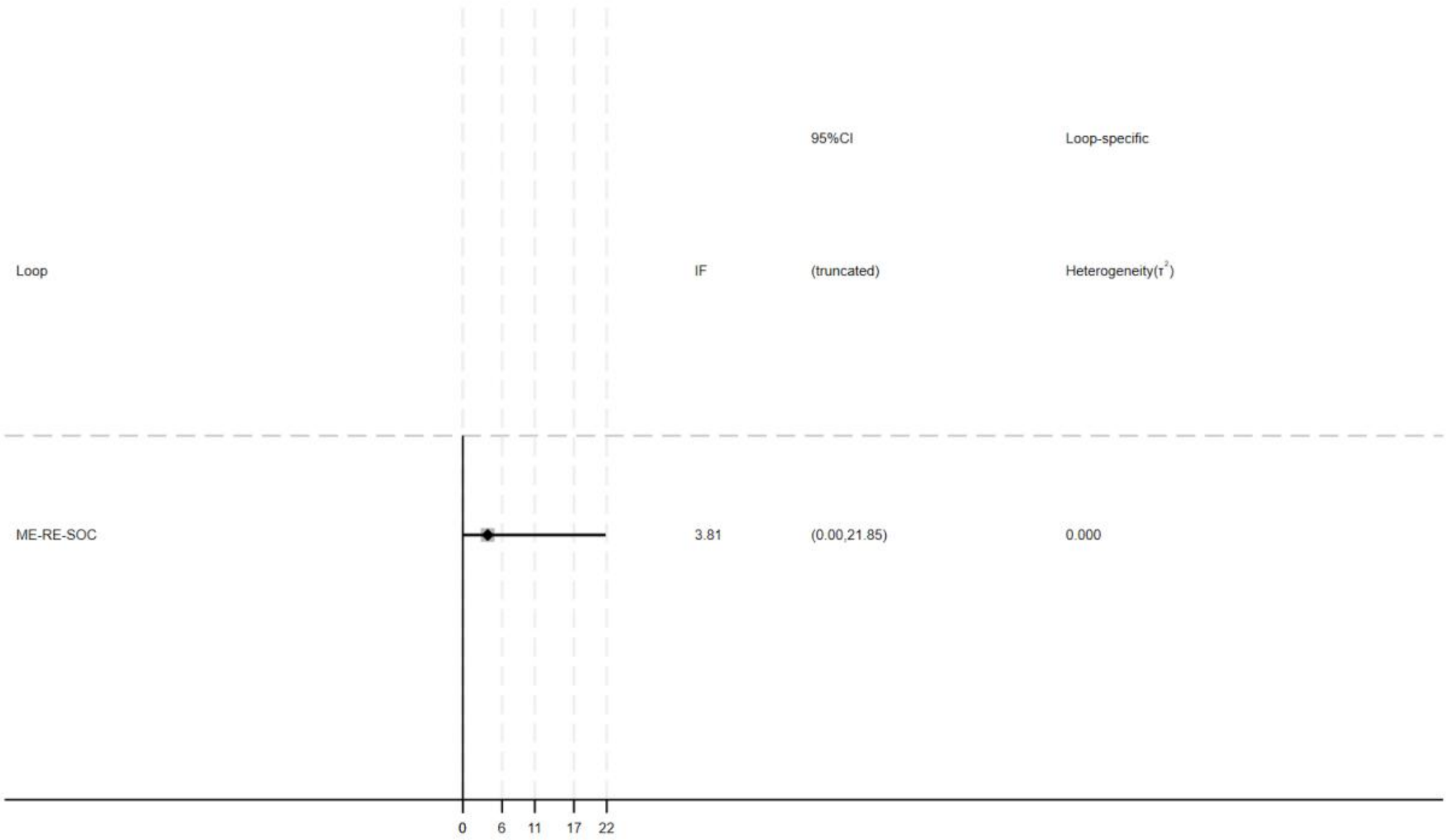

Supplementary Figure S1. Loop inconsistency plot for the SMI in adults with cancer

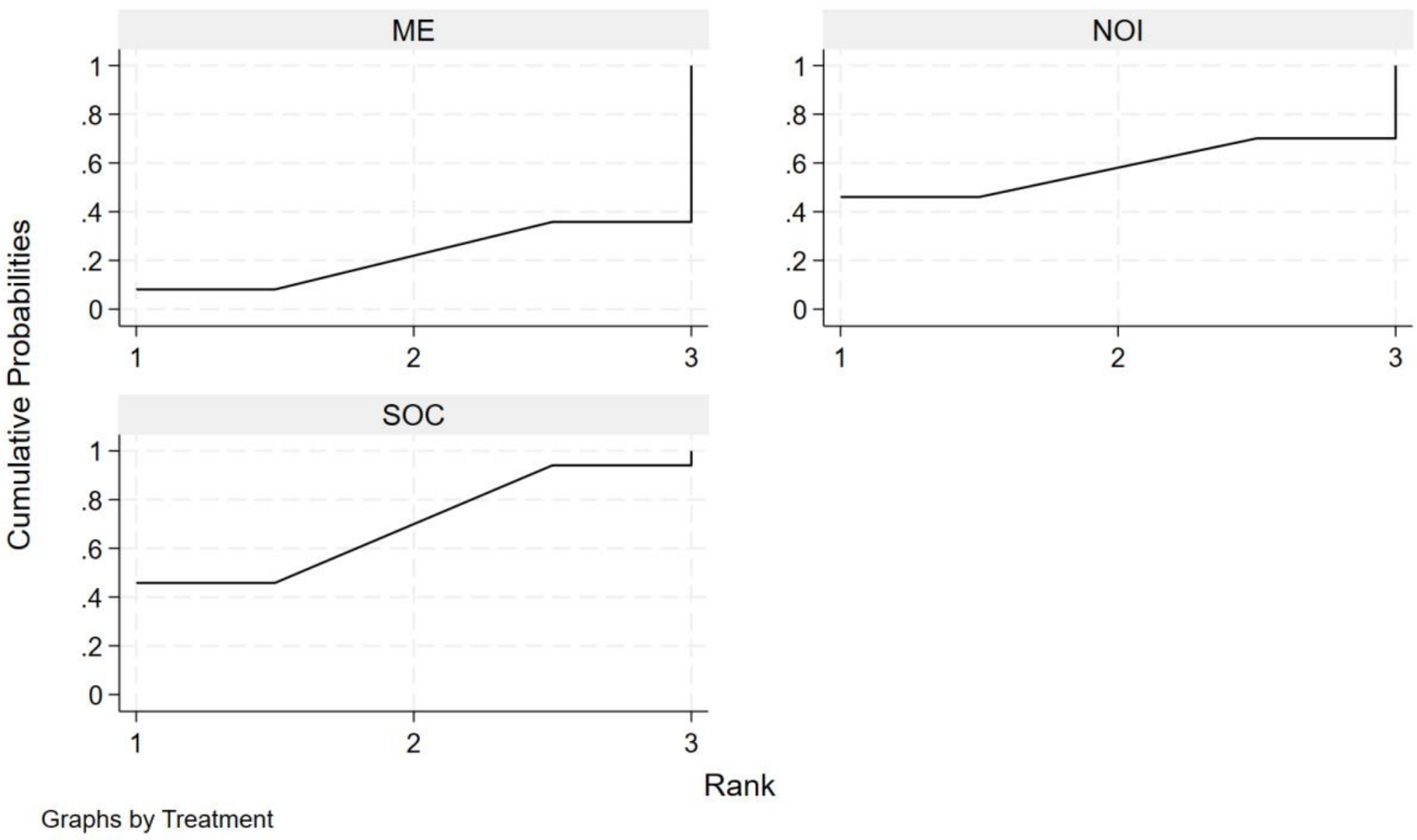

Supplementary Figure S2. Rank probability plot for the ASM outcome among

adults with cancer.

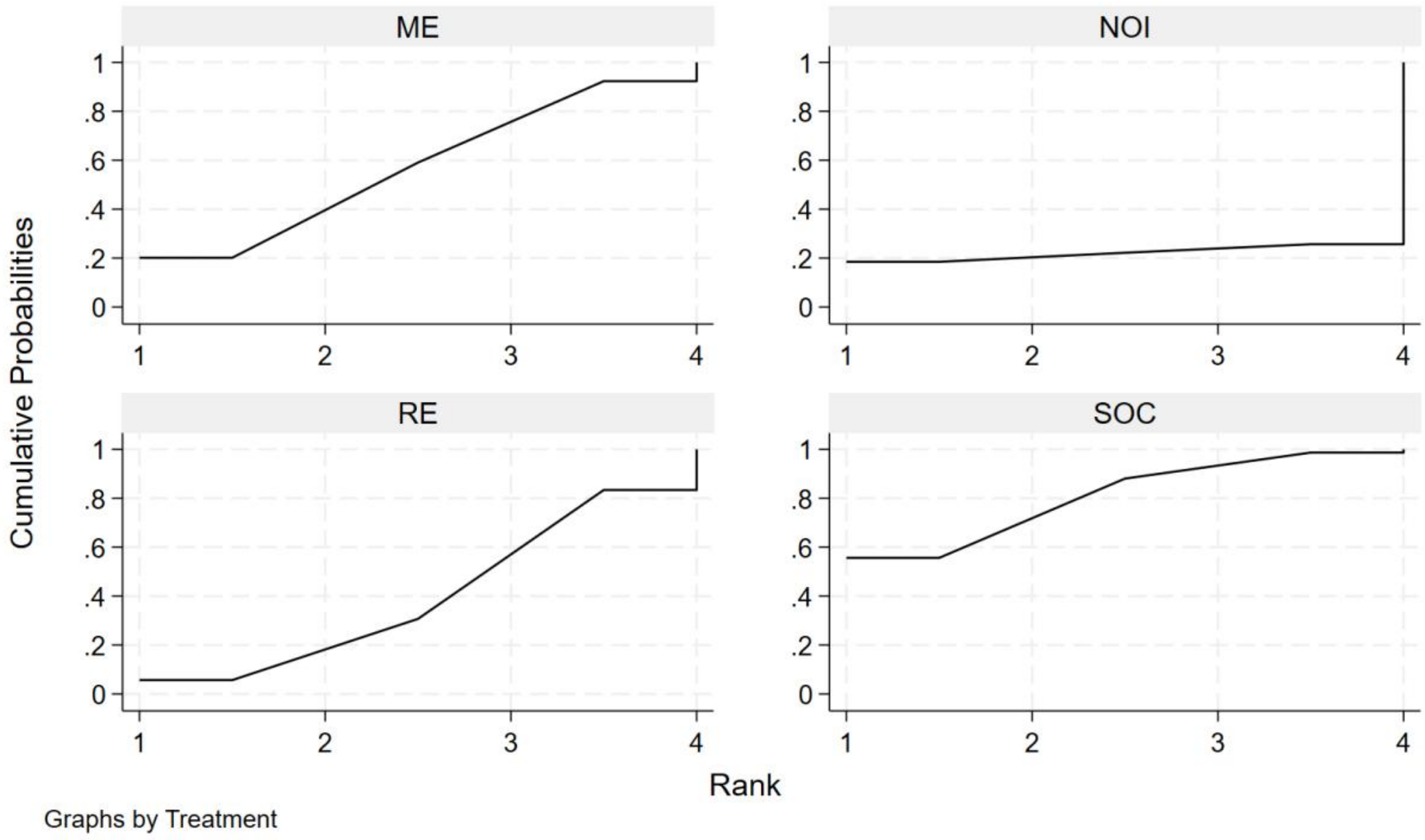

Supplementary Figure S3. Rank probability plot for the SMI outcome among adults with cancer.

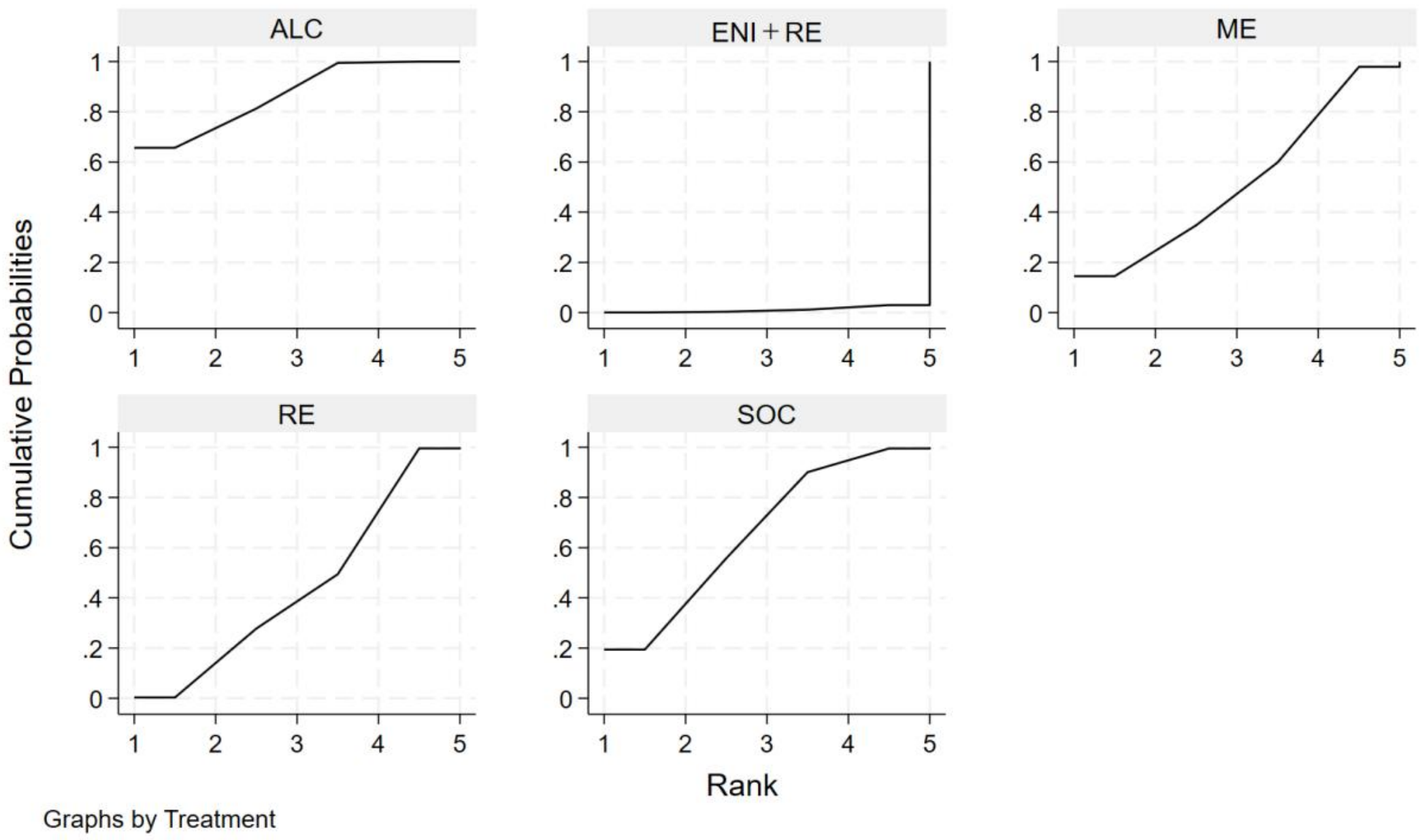

Supplementary Figure S4. Rank probability plot for the Lean mass outcome among

adults with cancer.

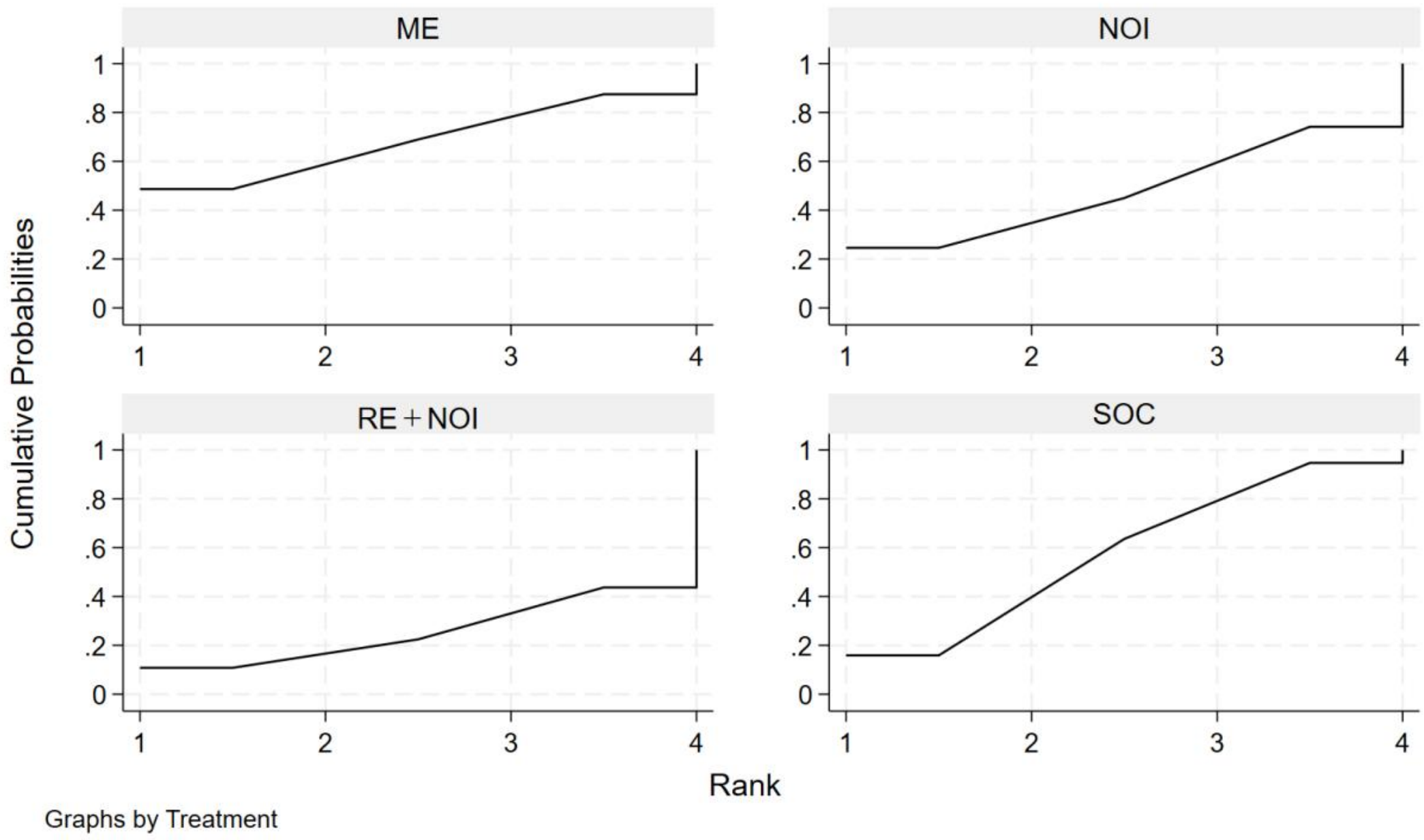

Supplementary Figure S5. Rank probability plot for the Body weight outcome among adults with cancer.

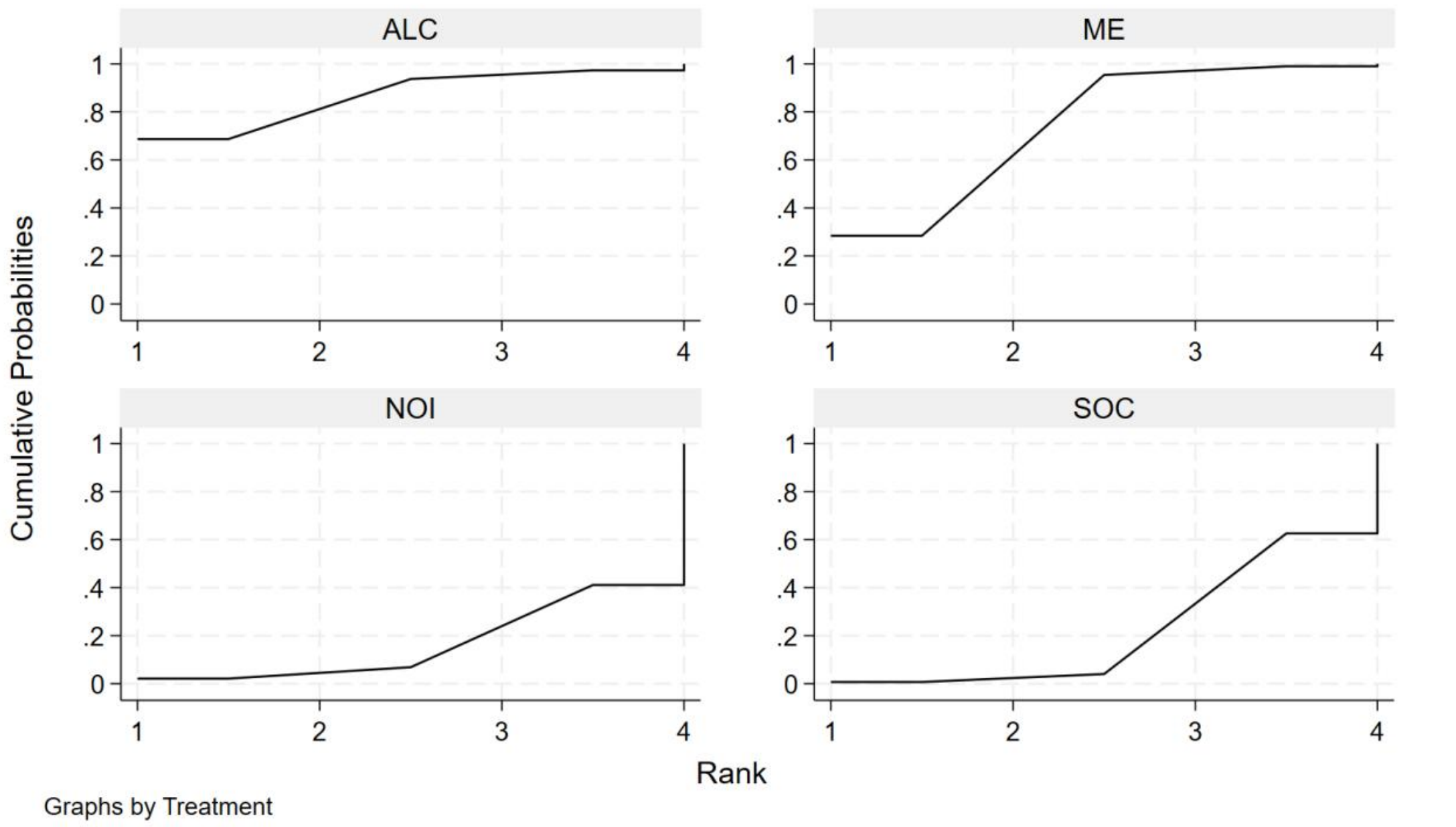

Supplementary Figure S6. Rank probability plot for the BMI outcome among adults with cancer.

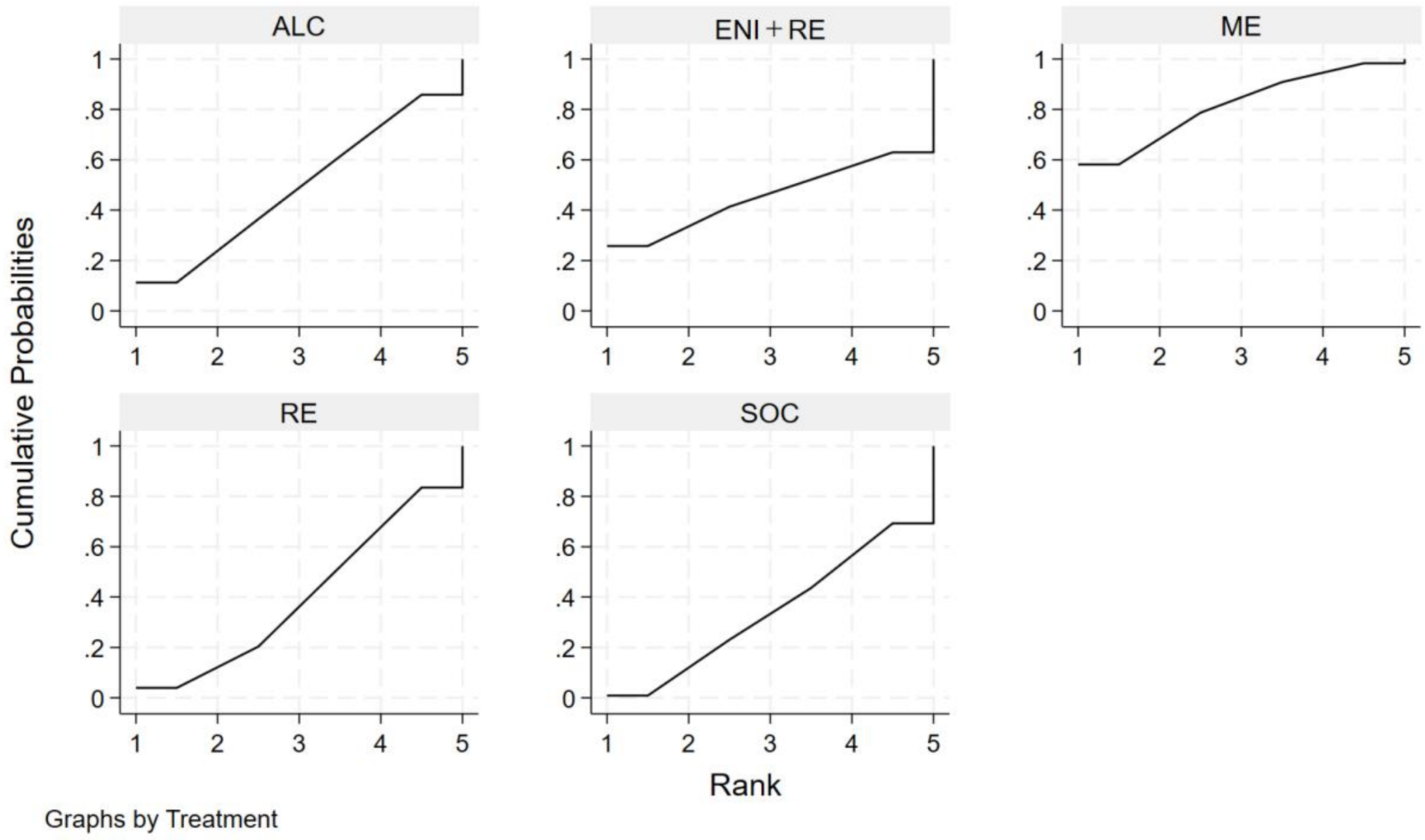

Supplementary Figure S7. Rank probability plot for the fat mass outcome among adults with cancer.

Supplementary Table S9. SUCRA ranking table for the ASM outcome in adults with cancer

Treatm~t	SUCRA	PrBest	MeanRank
SOC	70	36.6	1.6
NOI	58.1	46	1.8
ME	22	8.1	2.6

Supplementary Table S10. SUCRA ranking table for the SMI outcome in adults with cancer

Treatm~t	SUCRA	PrBest	MeanRank
SOC	80.8	55.6	1.6
ME	57.2	20.2	2.3
RE	39.9	5.7	2.8
NOI	22.1	18.5	3.3

Supplementary Table S11. SUCRA ranking table for the Lean mass outcome in adults with cancer

Treatm~t	SUCRA	PrBest	MeanRank
ALC	86.4	65.3	1.5

Treatm~t	SUCRA	PrBest	MeanRank
SOC	65.9	19.8	2.4
ME	52.4	14.5	2.9
RE	44.1	0.4	3.2
ENI + RE	1.2	0.1	5

Supplementary Table S12. SUCRA ranking table for the Body weight outcome in adults with cancer

Treatm~t	SUCRA	PrBest	MeanRank
ME	68.4	48.6	1.9
SOC	58.1	15.9	2.3
NOI	47.9	24.6	2.6
RE＋NOI	25.7	10.8	3.2

Supplementary Table S13. SUCRA ranking table for the BMI outcome in adults with cancer

Treatm~t	SUCRA	PrBest	MeanRank
ALC	86.4	68.6	1.4
ME	74.5	28.9	1.8
SOC	22.2	0.6	3.3
NOI	16.9	1.9	3.5

Supplementary Table S14. SUCRA ranking table for the Fat mass outcome in adults with cancer

Treatm~t	SUCRA	PrBest	MeanRank
SOC	34.2	0.9	3.6
RE	40	3.9	3.4
ENI + RE	45.6	25.8	3.2
ALC	48.7	11.3	3.1
ME	81.5	58.1	1.7

Supplementary Table S15. Sensitivity analysis for the Lean mass outcome in adults with

cancer

dropped_id	comparison	eff	lci	uci	connected
Manuel Grote2018	ALC vs SOC	-0.80000252	-4.522849	2.922843	1
Manuel Grote2018	ENI + RE vs SOC	6.7999961	0.6810772	12.91891	1
Manuel Grote2018	ME vs SOC	0.50818482	-1.721292	2.737661	1
Manuel Grote2018	RE vs SOC	0.79999798	-2.462154	4.06215	1
Dennis R Taaffe2019	ALC vs SOC	-1.4999435	-10.41428	7.414395	1
Dennis R Taaffe2019	ENI + RE vs SOC	6.1000556	-4.051152	16.25126	1
Dennis R Taaffe2019	ME vs SOC	0.50818589	-1.721265	2.737637	1
Dennis R Taaffe2019	RE vs SOC	0.10005567	-8.631935	8.832047	1
J Uth 2016	ALC vs SOC	-0.92819862	-6.131677	4.27528	1
J Uth 2016	ENI + RE vs SOC	6.6717996	-1.007887	14.35149	1
J Uth 2016	ME vs SOC	0.97689891	-2.545943	4.499741	1
J Uth 2016	RE vs SOC	0.67180223	-3.270105	4.613709	1
Dawson et al. 2021 (secondary analysis)	disconnected				0
Wendy Demark-Wahnefried2008	ALC vs SOC	-0.89199655	-4.696577	2.912584	1
Wendy Demark-Wahnefried2008	ENI + RE vs SOC	6.7080019	0.4540578	12.96195	1
Wendy Demark-Wahnefried2008	ME vs SOC	1.2805419	-1.601957	4.163041	1
Wendy Demark-Wahnefried2008	RE vs SOC	0.70800841	-2.485967	3.901984	1
Dawson et al. 2018 (primary report)	ALC vs SOC	-0.87761019	-4.076772	2.321552	1
Dawson et al. 2018 (primary report)	ENI + RE vs SOC	0.48440568	-1.560819	2.529631	1
Dawson et al. 2018 (primary report)	ME vs SOC	0.72238978	-2.16049	3.605269	1
Dieli-Conwright C.M. 2019	ALC vs SOC	-0.87761135	-4.076772	2.32155	1
Dieli-Conwright C.M. 2019	ENI + RE vs SOC	6.7223874	1.019591	12.42518	1
Dieli-Conwright C.M. 2019	ME vs SOC	-0.42163044	-2.76703	1.923769	1
Dieli-Conwright C.M. 2019	RE vs SOC	0.72238886	-2.16049	3.605268	1

Supplementary Table S16. Sensitivity analysis for the Body weight outcome in adults with cancer

dropped_id	comparison	eff	lci	uci	connected
Laviano A 2018	ME vs SOC	-3.3483965	-7.745677	1.048884	1

dropped_id	comparison	eff	lci	uci	connected
Laviano A 2018	NOI vs SOC	0.27369563	-1.706093	2.253484	1
Laviano A 2018	RE+NOI vs SOC	1.6	-0.6680655	3.868066	1
Galvão DA 2010	ME vs SOC	-0.76375889	-4.617958	3.09044	1
Galvão DA 2010	NOI vs SOC	1.9	-8.468292	12.26829	1
Galvão DA 2010	RE+NOI vs SOC	1.6	-3.114865	6.314865	1
Dawson et al. 2021 (secondary analysis)	ME vs SOC	-0.56317156	-4.127588	3.001245	1
Dawson et al. 2021 (secondary analysis)	NOI vs SOC	0.42617203	-3.287757	4.140101	1
Wendy Demark-Wahnefried2008	ME vs SOC	-0.64202992	-4.622455	3.338395	1
Wendy Demark-Wahnefried2008	NOI vs SOC	0.46580426	-3.64379	4.575399	1
Wendy Demark-Wahnefried2008	RE+NOI vs SOC	1.6	-2.947971	6.147971	1
Ritch C.R.2019	ME vs SOC	-0.76375889	-4.617958	3.09044	1
Ritch C.R.2019	NOI vs SOC	0.2	-4.4025	4.8025	1
Ritch C.R.2019	RE+NOI vs SOC	1.6	-3.114865	6.314865	1
Dieli-Conwright C.M. 2019	ME vs SOC	0.67495448	-0.3780744	1.727983	1
Dieli-Conwright C.M. 2019	NOI vs SOC	0.27369581	-1.706092	2.253484	1
Dieli-Conwright C.M. 2019	RE+NOI vs SOC	1.6	-0.6680655	3.868066	1

Supplementary Table S17. Sensitivity analysis for the BMI outcome in adults with cancer

dropped_id	comparison	eff	lci	uci	connected
Laviano A 2018	ALC vs SOC	-5.2390627	-7.37052	-3.107606	1
Laviano A 2018	ME vs SOC	-4.2390646	-6.119718	-2.358411	1
Laviano A 2018	NOI vs SOC	0.6	-2.194177	3.394177	1
Wendy Demark-Wahnefried2008	ALC vs SOC	-6.709981	-9.14606	-4.273902	1
Wendy Demark-Wahnefried2008	ME vs SOC	-5.7099834	-7.929945	-3.490021	1
Wendy Demark-Wahnefried2008	NOI vs SOC	0.22755441	-0.5058095	0.9609183	1
D'Silva C 2025	ALC vs SOC	-4.2390737	-6.119729	-2.358418	1
D'Silva C 2025	ME vs SOC	0.22755441	-0.5058095	0.9609183	1

dropped_id	comparison	eff	lci	uci	connected
Ritch C.R.2019	ALC vs SOC	-5.2390627	-7.37052	-3.107606	1
Ritch C.R.2019	ME vs SOC	-4.2390646	-6.119718	-2.358411	1
Ritch C.R.2019	NOI vs SOC	0.2	-0.5600079	0.9600079	1
Dieli-Conwright C.M. 2019	ALC vs SOC	-1.4999928	-5.178808	2.178823	1
Dieli-Conwright C.M. 2019	ME vs SOC	-0.49999315	-4.039405	3.039419	1
Dieli-Conwright C.M. 2019	NOI vs SOC	0.22755441	-0.5058095	0.9609183	1

Supplementary Table S18. Sensitivity analysis for the Fat mass outcome in adults with cancer

dropped_id	comparison	eff	lci	uci	connected
Manuel Grote2018	ALC vs SOC	-1.1999637	-8.828676	6.428749	1
Manuel Grote2018	ENI + RE vs SOC	-0.89996293	-12.3521	10.55218	1
Manuel Grote2018	ME vs SOC	-2.8640737	-6.720399	0.9922516	1
Manuel Grote2018	RE vs SOC	-0.69997471	-6.368908	4.968959	1
Dennis R Taaffe2019	ALC vs SOC	0.3999566	-9.101159	9.901073	1
Dennis R Taaffe2019	ENI + RE vs SOC	0.69995621	-12.07602	13.47593	1
Dennis R Taaffe2019	ME vs SOC	-2.8640738	-6.720444	0.9922963	1
Dennis R Taaffe2019	RE vs SOC	0.89996431	-7.113244	8.913173	1
J Uth 2016	ALC vs SOC	-0.91331824	-4.112225	2.285589	1
J Uth 2016	ENI + RE vs SOC	-0.61331793	-8.359727	7.133091	1
J Uth 2016	ME vs SOC	-4.8984925	-6.991426	-2.805559	1
J Uth 2016	RE vs SOC	-0.41331927	-3.124816	2.298177	1
Dawson et al. 2021 (secondary analysis)	disconnected				0
Wendy Demark-Wahnefried2008	ALC vs SOC	-0.68888087	-7.152888	5.775126	1
Wendy Demark-Wahnefried2008	ENI + RE vs SOC	-0.38888046	-10.92342	10.14566	1
Wendy Demark-Wahnefried2008	ME vs SOC	-3.2866017	-7.331056	0.7578525	1
Wendy Demark-Wahnefried2008	RE vs SOC	-0.18888117	-4.602923	4.225161	1
Dawson et al. 2018 (primary report)	ALC vs SOC	-0.72188847	-6.602339	5.158562	1
Dawson et al. 2018 (primary report)	ENI + RE vs SOC	-3.0448599	-6.503087	0.4133675	1
Dawson et al. 2018 (primary report)	ME vs SOC	-0.2218919	-4.324914	3.88113	1
Dieli-Conwright C.M. 2019	ALC vs SOC	-0.91331824	-4.112225	2.285589	1
Dieli-Conwright C.M. 2019	ENI + RE vs SOC	-0.61331793	-8.359727	7.133091	1
Dieli-Conwright C.M. 2019	ME vs SOC	-0.51080193	-4.174437	3.152833	1
Dieli-Conwright C.M. 2019	RE vs SOC	-0.41331927	-3.124816	2.298177	1

Supplementary Table S19. GRADE certainty of evidence for the ASM outcome in adults with cancer

Comparison	Number of studies	Within-study bias	Reporting bias	Indirectness	Imprecision	Heterogeneity	Incoherence	Confidence rating	Reason (s) for downgrading
SOC vs NOI	4	Some concerns	Low risk	No concerns	Some concerns	Some concerns	Not estimable	Low	["Within-study bias; Imprecision; Heterogeneity"]
SOC vs ME	4	Some concerns	Low risk	No concerns	Some concerns	Some concerns	Not estimable	Low	["Within-study bias; Imprecision; Heterogeneity"]
NOI vs ME	4	Some concerns	Low risk	No concerns	Some concerns	Some concerns	Not estimable	Low	["Within-study bias; Imprecision; Heterogeneity"]

Supplementary Table S20. GRADE certainty of evidence for the SMI outcome in adults

with cancer

Comparison	Number of studies	Within-study bias	Reporting bias	Indirectness	Imprecision	Heterogeneity	Incoherence	Confidence rating	Reason (s) for downgrading
SOC vs ME	3	Some concerns	Low risk	No concerns	Some concerns	No concerns	No concerns	Low	["Within-study bias","Imprecision"]
SOC vs RE	3	Some concerns	Low risk	No concerns	Some concerns	No concerns	No concerns	Low	["Within-study bias","Imprecision"]
SOC vs NOI	3	Some concerns	Low risk	No concerns	Some concerns	No concerns	No concerns	Low	["Within-study bias","Imprecision"]
ME vs RE	3	Some concerns	Low risk	No concerns	Some concerns	No concerns	No concerns	Low	["Within-study bias","Imprecision"]
ME vs NOI	3	Some concerns	Low risk	No concerns	Some concerns	No concerns	No concerns	Low	["Within-study bias","Imprecision"]
RE vs NO	3	Some concerns	Low risk	No concerns	Some concerns	No concerns	No concerns	Low	["Within-study bias","Imprecision"]

Supplementary Table S21. GRADE certainty of evidence for the Lean mass outcome in adults with cancer

Comparison	Number of studies	Within-study bias	Reporting bias	Indirectness	Imprecision	Heterogeneity	Incoherence	Confidence rating	Reason (s) for downgrading
ALC vs SOC	5	Some concerns	Low risk	Some concerns	Some concerns	No concerns	Not estimable	Low	["Within-study bias; Indirectness; Imprecision"]
ALC vs ME	5	Some concerns	Low risk	Some concerns	Some concerns	No concerns	Not estimable	Low	["Within-study bias; Indirectness; Imprecision"]
ALC vs RE	5	Some concerns	Low risk	Some concerns	Some concerns	No concerns	Not estimable	Low	["Within-study bias; Indirectness; Imprecision"]
ALC vs ENI+RE	5	No concerns	Low risk	Some concerns	Some concerns	No concerns	Not estimable	Low	["Within-study bias; Indirectness; Imprecision"]
SOC vs ME	5	No concerns	Low risk	Some concerns	Some concerns	No concerns	Not estimable	Low	["Within-study bias; Indirectness; Imprecision"]
SOC vs RE	5	No concerns	Low risk	Some concerns	Some concerns	No concerns	Not estimable	Low	["Within-study bias; Indirectness; Imprecision"]
SOC vs ENI+RE	5	No concerns	Low risk	Some concerns	Some concerns	No concerns	Not estimable	Low	["Within-study bias; Indirectness; Imprecision"]
ME vs RE	5	No concerns	Low risk	Some concerns	Some concerns	No concerns	Not estimable	Low	["Within-study bias; Indirectness; Imprecision"]
ME vs ENI+RE	5	No concerns	Low risk	Some concerns	Some concerns	No concerns	Not estimable	Low	["Within-study bias; Indirectness; Imprecision"]
RE vs ENI+RE	5	No concerns	Low risk	Some concerns	Some concerns	No concerns	Not estimable	Low	["Within-study bias; Indirectness; Imprecision"]

Supplementary Table S22. GRADE certainty of evidence for the Body weight outcome in adults with cancer

Comparison	Number of studies	Within-study bias	Reporting bias	Indirectness	Imprecision	Heterogeneity	Incoherence	Confidence rating	Reason (s) for downgrading
ME vs SOC	7	Some concerns	Low risk	Some concerns	Some concerns	Some concerns	Not estimable	Low	["Within-study bias; Indirectness; Imprecision; Heterogeneity"]
ME vs NOI	7	Some concerns	Low risk	Some concerns	Some concerns	Some concerns	Not estimable	Low	["Within-study bias; Indirectness; Imprecision; Heterogeneity"]
ME vs RE+NOI	7	Some concerns	Low risk	Some concerns	Some concerns	Some concerns	Not estimable	Low	["Within-study bias; Indirectness; Imprecision; Heterogeneity"]
SOC vs NOI	7	Some concerns	Low risk	Some concerns	Some concerns	Some concerns	Not estimable	Very low	["Within-study bias; Indirectness; Imprecision; Heterogeneity"]
SOC vs RE+NOI	7	Some concerns	Low risk	Some concerns	Some concerns	Some concerns	Not estimable	Moderate	["Within-study bias; Indirectness; Imprecision; Heterogeneity"]
NOI vs RE+NOI	7	Some concerns	Low risk	Some concerns	Some concerns	Some concerns	Not estimable	Moderate	["Within-study bias; Indirectness; Imprecision;

Comparison	Number of studies	Within-study bias	Reporting bias	Indirectness	Imprecision	Heterogeneity	Incoherence	Confidence rating	Reason (s) for downgrading
									Heterogeneity"]

Supplementary Table S23. GRADE certainty of evidence for the BMI outcome in adults with cancer

Comparison	Number of studies	Within-study bias	Reporting bias	Indirectness	Imprecision	Heterogeneity	Incoherence	Confidence rating	Reason (s) for downgrading
ALC vsME	6	Some concerns	Low risk	No concerns	Some concerns	Some concerns	Some concerns	low	["Within-study bias","Imprecision","Heterogeneity"
ALC vsSOC	6	Some concerns	Low risk	No concerns	Some concerns	Some concerns	Some concerns	low	["Within-study bias","Imprecision","Heterogeneity"
ALC vs NOI	6	Some concerns	Low risk	No concerns	Some concerns	Some concerns	Some concerns	Low	["Within-study bias","Imprecision","Heterogeneity"
ME vs SOC	6	Some concerns	Low risk	No concerns	Some concerns	Some concerns	Some concerns	Low	["Within-study bias","Imprecision","Heterogeneity"
ME vs NOI	6	Some concerns	Low risk	No concerns	Some concerns	Some concerns	Some concerns	Low	["Within-study bias","Imprecision","Heterogeneity"
SOC vs NOI	6	Some concerns	Low risk	No concerns	Some concerns	Some concerns	Some concerns	low	["Within-study bias","Imprecision","Heterogeneity"

Supplementary Table S24. GRADE certainty of evidence for the Fat mass outcome in adults with cancer

Comparison	Number of studies	Within-study bias	Reporting bias	Indirectness	Imprecision	Heterogeneity	Incoherence	Confidence rating	Reason (s) for downgrading
SOC vs RE	7	Some concerns	Low risk	No concerns	Some concerns	No concerns	No concerns	Low	["Within-study bias","Imprecision"]
SOC vs ENI+RE	7	Some concerns	Low risk	No concerns	Some concerns	No concerns	No concerns	Low	["Within-study bias","Imprecision"]
SOC vs ALC	7	Some concerns	Low risk	No concerns	Some concerns	No concerns	No concerns	Low	["Within-study bias","Imprecision"]
SOC vs ME	7	Some concerns	Low risk	No concerns	Some concerns	No concerns	Some concerns	Very low	["Within-study bias","Imprecision","Incoherence"]
RE vs ENI+RE	7	No concerns	Low risk	No concerns	Some concerns	No concerns	No concerns	Moderate	["Imprecision"]
RE vs ALC	7	No concerns	Low risk	No concerns	Some concerns	Some concerns	No concerns	Moderate	["Imprecision","Heterogeneity"]
RE vs ME	7	No concerns	Low risk	No concerns	Some concerns	No concerns	Major concerns	Very low	["Imprecision","Incoherence"]
ENI+RE vs ALC	7	No concerns	Low risk	No concerns	No concerns	Some concerns	Some concerns	Low	["Heterogeneity","Incoherence"]
ENI+RE vs ME	7	No concerns	Low risk	No concerns	Some concerns	No concerns	No concerns	Moderate	["Imprecision"]
ALC vs ME	7	No concerns	Low risk	No concerns	Some concerns	No concerns	No concerns	Moderate	["Imprecision"]

Supplementary Table S25. Clinical characteristics of control groups by outcome network

Panel A. Outcome-network characteristics and control-group heterogeneity

Outcome network	Contributing trials	Direct comparisons by node pair	Clinical and comparator variation
ASM	4	SOC–ME: 3; SOC–NOI: 1	Mixed, prostate, and head-and-neck cancers; active treatment, ADT, and chemoradiotherapy; placebo, usual care, delayed exercise, and walking-based active control.
SMI	3	SOC–ME: 2; SOC–NOI: 1; SOC–RE: 1; RE–ME: 1	Cervical, breast, esophagogastric, and bladder cancer reports; chemotherapy, neoadjuvant, and perioperative settings; placebo, usual care, usual pathway, or multivitamin controls.
Lean mass	5	SOC–ME: 2; SOC–RE: 1; RE–ALC: 1; ALC–ENI+RE: 1	Breast, head-and-neck, and prostate cancers; radiotherapy, chemotherapy, ADT, and post-treatment settings; usual care, delayed exercise, stretching, or dietary attention control.
Body weight	7	SOC–ME: 4; SOC–NOI: 2; SOC–RE+NOI: 1	Breast, prostate, lung, cervical, and bladder cancers; active treatment, ADT, cachexia, perioperative, and post-treatment settings; usual care, stretching, placebo, dietary attention, or multivitamin controls.
BMI	6	SOC–ME: 3; SOC–NOI: 2; ME–ALC: 1	Breast, cervical, head-and-neck, lung, and bladder cancers; chemotherapy, chemoradiotherapy, cachexia, perioperative, and post-treatment settings; active and placebo controls.
Fat mass	7	SOC–ME: 3; SOC–RE: 2; RE–ALC: 1; ALC–ENI+RE: 1	Breast, head-and-neck, and prostate cancers; radiotherapy, chemotherapy, ADT, and post-treatment settings; usual care, delayed exercise, stretching, or dietary attention control.

Interpretation: Control groups varied substantially in age, cancer site, treatment phase, and intervention intensity. Some controls received usual care only, whereas others received active exercise, dietary counselling, placebo drinks or capsules, or nutritional supplements. This heterogeneity may weaken the exchangeability assumption underlying indirect comparisons and was considered when judging transitivity and indirectness.

Panel B. Study-level inventory of control-group clinical characteristics

Study	Cancer type	Control n	Control age, years	Treatment/care setting	Control condition
Fu 2025	Mixed cancers	17	47.9±13.9	During treatment	Flavoured isocaloric placebo, 30 g three times/day
Galvão 2010	Prostate	28	70.1±7.3	Androgen-deprivation therapy (ADT)	Usual care; no structured exercise; customary activity/diet maintained
Lam 2020	Prostate	10	71.8±6.2	ADT	Usual care; non-standardized exercise advice; no structured programme
Dieli-Conwright 2019	Breast	49	50.8±11.0	After treatment	Usual care; exercise offered after study; usual dietary behaviour
Grote 2018	Head and neck	10	61.5±15.7	Radiotherapy	Usual care; inpatient physiotherapy permitted, without muscle strengthening
Taaffe 2019	Prostate	50	67.5±7.7	ADT	Delayed exercise/usual care for 6 months; calcium and vitamin D in all groups
Ford 2024	Colorectal	25	57±13	Chemotherapy	Standard protein target (1.0 g/kg/day), dietitian counselling, and weekly monitoring
Laviano 2020	Non-small-cell lung	29	66.0±8.0	Cancer cachexia / active treatment	Milk-based isocaloric comparator drink twice daily
Uth 2016	Prostate	28	66.5±4.9	ADT	Standard care; no structured exercise
Dawson trial (2018 primary; 2021 secondary analysis)	Prostate	19 in 2018 non-exercise analysis; 8 in 2021 subset	Primary report: group means; 2021 subset: median 65.5 [60.5, 72.5]	ADT	Protein supplementation and/or home-based stretching, depending on comparison; overlapping NCT01909440 reports
Demark-Wahnefried 2008	Breast	27	41.1±5.8	Adjuvant chemotherapy	Calcium-rich diet/attention control; no structured exercise or FVLF diet
Aredes 2019	Cervical	20	43.90±7.88	Chemoradiotherapy	Olive-oil placebo plus the same oral isocaloric nutritional supplement
Adams 2016	Breast	70	NR (overall 48.8; range 25–78)	Chemotherapy	Usual care; asked not to begin new exercise training
van der Werf 2020	Metastatic colorectal	54	66±10	First-line chemotherapy	Usual nutritional care; dietitian referral when clinically indicated

Study	Cancer type	Control n	Control age, years	Treatment/care setting	Control condition
D'Silva 2025	Head and neck	39	54.59±11.10	Chemoradiotherapy	Walking-based active control, 3×10 min/day, five days/week
Allen 2022	Esophagogastric	28	62±9	Neoadjuvant therapy before surgery	Usual pathway; encouraged aerobic activity; usual dietetic and psychological support
Burden 2017	Colorectal	45	68.9±11.49	Preoperative	Dietary advice alone; bottled water supplied for blinding
Fujimoto 2025	Gastrointestinal	33	80.6±5.4	Preoperative	Usual preoperative care; activity monitoring only
Ritch 2019	Bladder	24	67 (median)	Perioperative radical cystectomy	Multivitamin–multimineral supplementation twice daily
Uemura 2026	Pancreatic	30	71 [56–84] (median [range])	Chemotherapy	Normal diet; no supplement; standardized nutritional guidance
Anandavadivelan 2023	Esophageal	70	NR	After curative treatment	Usual care; encouraged to maintain routine daily physical activity

Abbreviations. ADT, androgen-deprivation therapy; ALC, active low-intensity control; ASM, appendicular skeletal muscle mass; BMI, body mass index; ENI, exercise–nutrition intervention; FVLF, fruit-and-vegetable, low-fat diet; ME, multicomponent exercise; NOI, nutrition-only intervention; NR, not reported; RE, resistance exercise; SMI, skeletal muscle index; SOC, standard of care.

Data note. Dawson et al. 2018 and Dawson et al. 2021 are companion reports from NCT01909440 and are treated as one randomized trial throughout the review.

Supplementary Table S26. Detailed intervention and control regimens

Detailed regimens are presented at the trial level. Dawson et al. 2018 and Dawson et al. 2021 are companion reports of the same randomized trial.

Study	Intervention arm	Control arm	Intervention intent	Nutrition therapy	Exercise prescription
**Jin Fu**	FSMP oral nutritional supplementation<br>Duration: 8 weeks;<br>Frequency: 3×/day<br>Intervention summary: Participants consumed 30 g of FSMP orally between meals each time.	Flavoured isocaloric placebo<br>Duration: 8 weeks;<br>Frequency: 3×/day<br>Control summary: Participants consumed 30g of flavoured isocaloric placebo orally between meals each time. The placebo provided 1812 kJ/100 g, 1.2 g protein/100 g, 15.4 g fat/100 g, and 82.0 g carbohydrate/100 g.	Nutritional status and muscle mass improvement during treatment	Frequency: Monthly;<br>Mode: face-to-face or telephone counselling;<br>Supplementation:<br>FSMP/placebo, 30 g, 3×/day, between meals	NR
**Galvão DA**	Combined resistance and aerobic exercise<br>Duration: 12 weeks;<br>Frequency: 2×/week<br>Intervention summary: Supervised small-group progressive resistance training plus low-volume aerobic exercise.	Usual careDuration: 12 weeks;<br>Frequency: N/A<br>Control summary: Participants received usual care without structured exercise training. They were encouraged to maintain customary activity and dietary patterns.	ADT-related muscle loss prevention	Total consultations: N/A<br>Frequency: N/A<br>Mode: N/A<br>Prescription: No nutrition intervention or dietary counselling was prescribed<br>Monitoring: Participants were encouraged to maintain customary dietary patterns; nutritional status was monitored using the Mini Nutritional Assessment	Type: Combined RE + AE, supervised, small-group-based<br>Frequency: 2×/week<br>Intensity: RE progressed from 12-RM to 6-RM; AE performed at 65%–80% maximum heart rate and Borg RPE 11–13<br>Volume: RE: 2–4 sets/exercise; AE: 15–20 min/session<br>Aim & progression: Progressive resistance training combined with low-volume aerobic exercise to improve muscle mass, strength, and physical function.
**Lam T**	Home-based progressive resistance training<br>Duration: 12 months / 52 weeks; Frequency: 3×/week<br>Intervention summary: Participants performed home-based PRT targeting all major muscle groups using adjustable dumbbells and body-weight loading.	Usual care<br>Duration: 12 months;<br>Frequency: N/A<br>Control summary: Participants received usual care. As part of routine care, they received non-standardized advice from their treating clinician to participate in regular exercise, but no structured exercise programme was provided.	ADT-related body composition maintenance	Total consultations: N/A<br>Frequency: N/A<br>Mode: N/A<br>Prescription: No nutrition intervention or dietary counselling was prescribed<br>Monitoring: NR	Type: PRT, home-based, partially supervised<br>Frequency: 3×/week<br>Intensity: 8–12 RM loading<br>Volume: 8–10 exercises targeting major muscle groups; 3 sets/exercise<br>Aim & progression: Individualized progression in loading or difficulty when participants could complete >12 repetitions per set; participants progressed through three exercise stages according to confidence and ability<br>Support: Two supervised sessions at baseline, one supervised session every 12 weeks, online instructional videos, printed training manual, monthly reminder phone calls, and exercise logbook.

Study	Intervention arm	Control arm	Intervention intent	Nutrition therapy	Exercise prescription
**Dieli-Conwright CM**	Aerobic and resistance exercise Duration: 16 weeks;<br>Frequency: 3×/week;<br>Intervention summary: supervised combined moderate-to-vigorous aerobic and resistance exercise.	Usual care Duration: 16 weeks;<br>Frequency: N/A<br>Control summary:Participants continued usual care and were offered the exercise programme after the study period.	Weight loss and body composition improvement after treatment	Total consultations: N/A<br>Frequency: N/A<br>Mode: N/A<br>Prescription: No nutrition intervention or dietary counselling was prescribed. Participants were asked to maintain usual dietary behaviours.<br>Monitoring: Three-day dietary records were completed and analysed.	Type: Combined AE + RE, supervised, clinical, one-on-one.<br>Frequency: 3×/week.<br>Intensity: Aerobic exercise at 65%–80% HRmax; warm-up/cool-down at 40%–50% estimated VO_2max. Resistance exercise started at ~80% estimated 1-RM for lower-body exercises and 60% estimated 1-RM for upper-body exercises.<br>Volume: 150 min/week aerobic exercise plus 2–3 days/week resistance training was promoted. Aerobic duration increased from 30 min to 50 min. Resistance repetitions progressed from 10 to 12 to 15 every 4 weeks; load increased by 10% when participants completed 3 sets of 10 repetitions in 2 consecutive sessions.
**Grote M**	Intervention summary:<br>Participants performed home-based PRT targeting all major muscle groups using adjustable dumbbells and body-weight loading.	Usual care Duration: during radiotherapy;<br>Frequency: N/A;<br>Control summary: Participants received usual care. Inpatient physiotherapy could be prescribed by the ward physician, but muscle-strengthening techniques were not part of usual care.	Muscle mass maintenance during radiotherapy	Total consultations: N/A<br>Frequency: N/A<br>Mode: N/A<br>Prescription: No nutrition intervention or dietary counselling was prescribed.<br>Monitoring: NR	Type: PRT, machine-supported, supervised.<br>Frequency: planned 3×/week; actual mean 2.7×/week. Intensity: 8–12 RM; RPE monitored after each machine; loading increased if RPE < 7.<br>Volume: 30 min/session; 3 exercises, 3 sets each, 8–12 repetitions/set; maximum 60-s rest between sets/exercises. Progression: +5.0 kg for lower-limb exercise and +2.5 kg for upper-limb exercises when tolerated.
**Taaffe DR**	Combined resistance, aerobic and impact-loading exercise Duration: 6 months;<br>Frequency: 3×/week<br>Intervention summary: Participants received supervised clinic-based multicomponent exercise from the onset of ADT. The programme combined resistance training, aerobic exercise, and impact-loading activities.	Delayed exercise / usual care Duration: 6 months usual care, followed by 6 months identical exercise programme;<br>Frequency: N/A during first 6 months<br>Control summary: Participants received usual care during the first 6 months and then undertook the same resistance, aerobic, and impact-loading exercise programme during months 7–12.	ADT-related muscle and fat mass maintenance	Total consultations: N/A<br>Frequency: Daily supplementation<br>Mode: Oral supplementation<br>Prescription: All participants received standard calcium 1000 mg/day and vitamin D3 800 IU/day. No nutrition counselling or dietary intervention was prescribed.<br>Monitoring: NR	Type: Combined RE + AE + impact-loading exercise, supervised, small-group clinic-based.<br>Frequency: 3×/week supervised; participants were also encouraged to perform 2×/week home-based aerobic and modified impact-loading exercise.<br>Intensity: Resistance training at 6–12 RM; aerobic exercise at 60%–85% estimated maximum heart rate; impact loading produced peak ground reaction forces of 3.4–5.2× body weight.<br>Volume: ~60 min/session; resistance training 2–4 sets/exercise; aerobic exercise 25–40 min/session. Exercise sessions

Study	Intervention arm	Control arm	Intervention intent	Nutrition therapy	Exercise prescription
					alternated weekly between aerobic/impact and resistance/impact sessions.
**Ford KL**	Standard-protein dietary intervention Duration: 12 weeks; Frequency: Daily<br>Control summary: Participants were instructed to achieve 1.0 g/kg/day protein intake, representing the minimum standard of care.	Standard-protein dietary intervention Duration: 12 weeks;<br>Frequency: Daily<br>Control summary: Participants were instructed to achieve 1.0 g/kg/day protein intake, representing the minimum standard of care. They also received individualized nutrition counselling and weekly dietary monitoring.	Muscle mass maintenance during chemotherapy	Total consultations:<br>Baseline individualized dietitian counselling + 6-week follow-up<br>Frequency: Weekly telephone contact; dietary assessment at baseline, week 6, and week 12<br>Mode: Individualized face-to-face nutrition counselling and telephone monitoring<br>Prescription: 2.0 g/kg/day protein in the intervention group; 1.0 g/kg/day protein in the control group; daily multivitamin provided. Whey protein powder was provided if protein targets were not met.<br>Monitoring: 3-day weighed food records and weekly 24-h dietary recalls; nutrition-impact symptoms and self-reported body weight were also monitored.	NR
**Laviano et al. 2020**	Targeted medical nutrition<br>Duration: 12 weeks; Frequency: 2×/day<br>Intervention summary: Participants received juice-based TMN, 200 mL/package, twice daily.	Isocaloric comparator drink<br>Duration: 12 weeks;<br>Frequency: 2×/day;<br>Control summary: Participants received a milk-based isocaloric comparator drink, 200 mL/package, twice daily. It provided similar energy but did not contain DHA/EPA fish oil or vitamin D3.	Weight and lean mass maintenance in cachexia	Frequency: 2×/day oral supplementation<br>Mode: Ready-to-drink oral nutritional supplement<br>Consultations: NR<br>Monitoring: Drink consumption diary was used to assess compliance.	NR
**Uth J**	Football training<br>Duration: 32 weeks;<br>Frequency: 2–3×/week<br>Intervention summary: Participants performed recreational football training for 32 weeks.	Standard care<br>Duration: 32 weeks<br>Control summary:<br>Participants received standard care and did not receive structured exercise training during the intervention period.	ADT-related body composition maintenance	NR	Type: Recreational football training, supervised/group-based<br>Frequency: 2–3×/week<br>Intensity: Variable intermittent intensity with football-specific accelerations, decelerations, and directional changes<br>Volume: 45–60 min/session; 32 weeks
**Dawson et al. 2018/2021**	Periodized resistance training<br>Duration: 12 weeks;	Home-based stretching<br>Duration: 12 weeks;	ADT-related muscle mass improvement	Total consultations: N/A<br>Frequency: Weekly dietary records	Type: Periodized resistance training, supervised, one-on-one<br>Frequency: 3×/week

Study	Intervention arm	Control arm	Intervention intent	Nutrition therapy	Exercise prescription
	Frequency: 3×/week<br>Intervention summary: Participants performed supervised one-on-one resistance training.	Frequency: 3×/week<br>Control summary: Participants performed a home-based flexibility program lasting ~5 min/session using stretching exercises similar to those performed at the end of the exercise group's sessions.		Mode: Dietary intake records reviewed; some participants received protein supplementation<br>Prescription: Participants not meeting 0.8 g/kg/day protein intake were instructed to increase protein intake. Protein intake did not differ significantly between EXE and STRETCH groups.	Intensity: 60%–83% 1RM<br>Volume: ~50 min/session; 3 sets/exercise; 12 weeks<br>Progression: Weeks 1–2: 60% 1RM, 15 reps; Weeks 3–4: 65%–67% 1RM, 15–12 reps; Weeks 5–6: 70% 1RM, 12–10 reps; Weeks 7–8: 75% 1RM, 10–8 reps; Weeks 9–10: 80% 1RM, 10–8 reps; Weeks 11–12: 83% 1RM, 8 reps.
**Demark-Wahnefried W**	Home-based diet and exercise intervention Duration: 6 months; Frequency: aerobic exercise ≥3×/week; strength training every other day Intervention summary:CA+EX received a calcium-rich diet plus home-based aerobic and strength-training exercises. CA+EX+FVLF received the same calcium-rich diet and exercise intervention plus a high fruit and vegetable, low-fat diet.	Calcium-rich diet / attention<br>control Duration: 6 months;<br>Frequency: NR for diet intake; telephone counselling schedule same as intervention arms Control summary: Participants received written and verbal instruction on a calcium-rich diet with target intake of 1200–1500 mg/day calcium. No structured exercise or FVLF diet was prescribed.	Weight gain prevention during treatment	Total consultations: 14 telephone counselling contacts<br>Frequency: once weekly for the first month, then semiweekly for the remaining 5 months<br>Mode: mailed materials + telephone counselling<br>Prescription: All groups received calcium-rich diet advice targeting 1200–1500 mg/day calcium. CA+EX+FVLF additionally targeted ≤20% energy from fat and ≥5 servings/day fruit and vegetables.<br>Monitoring: self-monitoring logs and dietary questionnaires.	Type: Home-based AE + strength<br>training.<br>Delivery: mailed workbook, videotape, telephone counselling, headset, pace tapes, heart-rate monitor, exercise ball, resistance bands, and water-fillable ankle weights.<br>Frequency: aerobic exercise ≥30 min/day, ≥3×/week; strength training every other day.<br>Intensity: moderate-to-high intensity aerobic exercise promoted using pace tapes and heart-rate monitors; strength training used body weight, therabands, and ankle weights.<br>Volume: 7 strength exercises, lower-body focused; 2 sets of stretches to avoid injury.
**Aredes MA**	Omega-3 supplementation<br>Duration: 45 days;<br>Frequency: 4 capsules/day<br>Intervention summary: Participants received omega-3 supplementation totaling 2.5 g/day, including 2 g EPA and 450 mg DHA.	Olive oil placebo Duration:<br>45 days;<br>Frequency: 4 capsules/day<br>Control summary: Participants received identical-looking placebo capsules containing olive oil. They also received the same oral isocaloric nutritional supplement as the intervention group.	Nutritional status and skeletal muscle quality maintenance during treatment	Total consultations: T0 and<br>T1 research/nutrition assessment visits<br>Frequency: Daily supplementation for 45 days; weekly capsule-adherence monitoring<br>Mode: Oral capsules + oral powdered nutritional supplement<br>Prescription: IG received omega-3 2.5 g/day; both groups received oral isocaloric nutritional supplement providing 430 kcal/day and 16 g protein/day.<br>Monitoring: Weekly capsule-intake evaluation, returned capsule count, and baseline 3-day food registry.	NR
**Adams SC**	Resistance exercise<br>training and aerobic exercise training Duration:	Usual care Duration: during<br>chemotherapy;	Sarcopenia and dynapenia improvement during	NR	Type: RET or AET, supervised<br>exercise training.

Study	Intervention arm	Control arm	Intervention intent	Nutrition therapy	Exercise prescription
	during chemotherapy, median 17 weeks; Frequency: 3×/week<br>Intervention summary: Participants were assigned to either RET or AET.	Frequency: N/A<br>Control summary: Participants were asked to avoid beginning any new exercise training during treatment and were invited to participate in a 1-month supervised exercise programme after postintervention testing.	chemotherapy		Delivery: supervised sessions during chemotherapy. Frequency: 3×/week.<br>Intensity: RET at 60%–70% predicted 1-RM; AET included 15 min at 60% VO_2peak and 45 min at 80% VO_2peak.<br>Volume: RET: 2 sets of 8–12 repetitions for 9 exercises; AET: 60 min/session.
**van der Werf A**	Individualized nutritional counseling Duration: during first-line chemotherapy; mean 9±3 weeks until T1 and 19±3 weeks until T2<br>Frequency: face-to-face at every chemotherapy cycle plus telephone consultations between cycles Intervention summary: Participants received individualized nutritional counseling by a trained study dietitian.	Usual nutritional care<br>Duration: during first-line chemotherapy<br>Frequency: as clinically indicated<br>Control summary: Participants received usual care and were referred to a regular care dietitian when indicated by shared decision-making between the patient and treating oncologist.	Muscle mass maintenance during chemotherapy	Total consultations: NC mean 12.1±4.6 consultations; UC patients receiving counseling mean 3.5±2.5 consultations<br>Frequency: face-to-face counseling at every chemotherapy cycle; telephone consultations between cycles<br>Mode: individualized face-to-face and telephone dietitian counseling<br>Prescription: protein target 1.2 g/kg/day, preferably 25 g protein per meal; energy requirement calculated by Harris–Benedict equation plus 30%; energy- and protein-enriched diet recommended; oral nutritional supplements prescribed if intake was <75% of goals or significant weight loss occurred; tube feeding if intake remained insufficient.<br>Monitoring: food diary and dietary intake assessment; physical activity monitored with accelerometer at study time points.	Type: N/A; no structured exercise intervention.<br>Delivery: physical activity advice only<br>Frequency: encouraged 5×/week Intensity: moderate intensity.<br>Volume: 30 min/day.<br>Activity examples: walking, cycling, or swimming.
**D'Silva C**	Combined aerobic and resistance training Duration:7 weeks; Frequency:5×/week<br>Intervention summary:Participants received structured exercise-based rehabilitation during chemoradiotherapy.	Walking-based exercise<br>Duration:7weeks;<br>Frequency:5×/week;<br>Control summary:Participants were advised to walk 3×10 min/day, 5 days/week, according to NCCN guideline recommendations, with intensity maintained at 3–5/10 on the modified Borg scale.	Sarcopenia prevention during chemoradiotherapy	NR	Type:Combined AE + RE, hospital-based during chemoradiotherapy.<br>Frequency:5×/week, except on chemotherapy day.<br>Intensity:3–5/10 RPE on modified Borg scale.<br>Volume:15–20 min/session; resistance training included 2 sets, 8–15 repetitions/set, using resistance bands for upper- and lower-limb muscles. Aerobic component:brisk walking.

Study	Intervention arm	Control arm	Intervention intent	Nutrition therapy	Exercise prescription
**Allen et al. 2022**	Multimodal prehabilitation Duration:15 weeks; Frequency:supervised 2×/week + home exercise 3×/week<br>Intervention summary:Participants received a multimodal prehabilitation programme during neoadjuvant therapy before esophagogastric cancer surgery.	Usual care Duration:during neoadjuvant therapy before surgery;<br>Frequency:N/A<br>Control summary:Participants followed the standard esophagogastric cancer care pathway. They were encouraged to remain active through regular aerobic activity such as walking, jogging, or cycling, and received usual dietetic input and nurse-led psychological support.	Muscle mass and fitness maintenance during neoadjuvant therapy	Total consultations:NR<br>Frequency:frequent, tailored dietetic input<br>Mode:specialist dietitian support Prescription:All patients received needs-based nutritional intervention, with increased calorie and protein intake where appropriate according to assessment and activity level.<br>Monitoring:NR	Type:Combined AE + RE + flexibility<br>training, supervised and home-based. Frequency:supervised 2×/week; home exercise 3×/week. Intensity:Aerobic cycling progressed from 40% to 60% HRR, RPE 11–14/20; resistance exercise at RPE 12–14/20.<br>Volume:Supervised sessions 1 h/session for 15 weeks; aerobic cycling 25 min/session including 5-min warm-up and 20-min training; resistance exercises for 6 major muscle groups, 2 sets × 12 repetitions; home exercise 1 h/session using resistance band and core stability exercises.
**Burden ST**	Oral nutritional<br>supplementation plus dietary advice Duration: pre-operative period, median 8 days; Frequency: 250 mL/day<br>Intervention summary: Participants received oral nutritional supplements from allocation until the day before surgery, together with dietary advice aimed at increasing energy and protein intake.	Dietary advice alone Duration:<br>pre-operative period;<br>Frequency: N/A<br>Control summary: Participants received the same dietary advice leaflet and structured discussion with a nutritionist, but did not receive active oral nutritional supplements. For blinding, they received sealed boxes containing bottled water.	Preoperative weight loss prevention	Total consultations: baseline<br>nutritionist discussion.<br>Frequency: daily ONS until the day before surgery; median provision 8 days Mode: oral nutritional supplement plus dietary advice leaflet and structured dietary discussion.<br>Prescription: 250 mL/day Fortisip Compact; dietary advice encouraged high-energy and high-protein foods.<br>Monitoring: drink consumption diary, 24-h dietary recall, body weight, handgrip strength, and BIA-based body composition.	NR
**Fujimoto T**	Preoperative resistance<br>training and amino acid supplementation Duration:14 days;<br>Frequency:daily amino acid supplementation + resistance exercise 2×/day<br>Intervention summary:Participants performed home-based resistance exercises and consumed amino acid-containing jelly daily before surgery.	Usual preoperative care / activity monitoring Duration:14 days before surgery;<br>Frequency:N/A<br>Control summary:Participants did not receive resistance training or amino acid supplementation. Both groups wore an activity tracker for 2 weeks before surgery.	Preoperative physical function maintenance	Total consultations:N/A<br>Frequency:once daily for 14 days<br>Mode:oral amino acid-containing jelly<br>Prescription:Participants consumed Amino L40 jelly once daily; the supplement contained 40% leucine.<br>Monitoring:NR for nutrition adherence; both groups wore an activity tracker.	Type:Home-based bodyweight<br>resistance training.<br>Delivery:home-based, self-recorded using a training log.<br>Frequency:2×/day for 14 days.<br>Intensity:bodyweight exercise; exact intensity NR.<br>Volume:four exercises: 20 seated knee extensions, 20 standing thigh lifts, 10 standing calf raises, and 5–10 squats per session.<br>Adherence:mean exercise adherence rate 85.2%.
**Ritch CR**	Enriched oral nutrition<br>supplementation Duration:3–4 weeks before	Multivitamin multimineral<br>supplementation Duration:3–4 weeks before	Postoperative sarcopenia prevention	Total consultations:NR<br>Frequency:supplement delivery every other week;	NR

Study	Intervention arm	Control arm	Intervention intent	Nutrition therapy	Exercise prescription
	radical cystectomy and 4 weeks after surgery;<br>Frequency:2×/day<br>Intervention summary:Participants received an enriched oral nutrition supplement, Ensure Clinical Strength, twice daily during the perioperative period.	radical cystectomy and 4 weeks after surgery;<br>Frequency:2×/day<br>Control summary:Participants received a standard oral multivitamin multimineral supplement twice daily during the same perioperative period.		supplementation 2×/day Mode:oral nutritional supplement; clinic-based follow-up with research dietitian<br>Prescription:ONS group received Ensure Clinical Strength twice daily; control group received multivitamin multimineral supplement twice daily.<br>Monitoring:weekly supplement-use checklist reviewed by a registered dietitian nutritionist; dietary intake assessed by 24-h recall.	
**Uemura S**	Elemental diet supplementation<br>Duration:at least 2 months; median duration 5.6 months;<br>Frequency:1 packet/day<br>Intervention summary:Participants received oral elemental diet supplementation during chemotherapy.	Normal diet Duration:during chemotherapy;<br>Frequency:N/A<br>Control summary:Participants received normal diet only. No nutritional supplements were provided. Both groups received standardized nutritional guidance during chemotherapy.	Nutritional status preservation during chemotherapy	Total consultations:NR<br>Frequency:standardized nutritional guidance every 2 weeks; ED 1 packet/day in the intervention group<br>Mode:oral elemental diet supplementation + nutritional guidance Prescription:EI group received Elental 1 packet/day for at least 2 months; ND group received no nutritional supplements.<br>Monitoring:serum nutritional markers, BIA-based body composition, CT-derived SMI and ATI, and monthly handgrip strength; individual ED adherence data were not collected.	NR
**Anandavadivelan P**	Home-based physical activity program. Duration: 12 weeks.<br>Frequency: target 150 min/week physical activity;<br>Intervention summary: Patients were prescribed a tailored home-based physical activity intervention after curative treatment for esophageal cancer.	Usual care Duration: 12 weeks;<br>Frequency: N/A;<br>Control summary: Participants were encouraged to maintain routine daily physical activity.	Muscle strength and muscle mass improvement after esophageal cancer treatment	NR	Type: home-based physical activity, including aerobic activity and simple strength exercises.<br>Delivery: home-based.<br>Frequency: target 150 min/week physical activity; strength exercises twice weekly. Intensity: minimum-intensity activities such as walking, swimming, or cycling. Volume: 150 min/week physical activity; 5 simple strength-training exercises.
